# The *Sorcerer II* Global Ocean Sampling Expedition: Expanding the Universe of Protein Families

**DOI:** 10.1371/journal.pbio.0050016

**Published:** 2007-03-13

**Authors:** Shibu Yooseph, Granger Sutton, Douglas B Rusch, Aaron L Halpern, Shannon J Williamson, Karin Remington, Jonathan A Eisen, Karla B Heidelberg, Gerard Manning, Weizhong Li, Lukasz Jaroszewski, Piotr Cieplak, Christopher S Miller, Huiying Li, Susan T Mashiyama, Marcin P Joachimiak, Christopher van Belle, John-Marc Chandonia, David A Soergel, Yufeng Zhai, Kannan Natarajan, Shaun Lee, Benjamin J Raphael, Vineet Bafna, Robert Friedman, Steven E Brenner, Adam Godzik, David Eisenberg, Jack E Dixon, Susan S Taylor, Robert L Strausberg, Marvin Frazier, J. Craig Venter

**Affiliations:** 1 J. Craig Venter Institute, Rockville, Maryland, United States of America; 2 University of California, Davis, California, United States of America; 3 Razavi-Newman Center for Bioinformatics, Salk Institute for Biological Studies, La Jolla, California, United States of America; 4 Burnham Institute for Medical Research, La Jolla, California, United States of America; 5 University of California Los Angeles–Department of Energy Institute for Genomics and Proteomics, Los Angeles, California, United States of America; 6 University of California Berkeley, Berkeley, California, United States of America; 7 Physical Biosciences Division, Lawrence Berkeley National Laboratory, Berkeley, California, United States of America; 8 University of California San Diego, San Diego, California, United States of America; 9 Brown University, Providence, Rhode Island, United States of America; Washington University St. Louis, United States of America

## Abstract

Metagenomics projects based on shotgun sequencing of populations of micro-organisms yield insight into protein families. We used sequence similarity clustering to explore proteins with a comprehensive dataset consisting of sequences from available databases together with 6.12 million proteins predicted from an assembly of 7.7 million Global Ocean Sampling (GOS) sequences. The GOS dataset covers nearly all known prokaryotic protein families. A total of 3,995 medium- and large-sized clusters consisting of only GOS sequences are identified, out of which 1,700 have no detectable homology to known families. The GOS-only clusters contain a higher than expected proportion of sequences of viral origin, thus reflecting a poor sampling of viral diversity until now. Protein domain distributions in the GOS dataset and current protein databases show distinct biases. Several protein domains that were previously categorized as kingdom specific are shown to have GOS examples in other kingdoms. About 6,000 sequences (ORFans) from the literature that heretofore lacked similarity to known proteins have matches in the GOS data. The GOS dataset is also used to improve remote homology detection. Overall, besides nearly doubling the number of current proteins, the predicted GOS proteins also add a great deal of diversity to known protein families and shed light on their evolution. These observations are illustrated using several protein families, including phosphatases, proteases, ultraviolet-irradiation DNA damage repair enzymes, glutamine synthetase, and RuBisCO. The diversity added by GOS data has implications for choosing targets for experimental structure characterization as part of structural genomics efforts. Our analysis indicates that new families are being discovered at a rate that is linear or almost linear with the addition of new sequences, implying that we are still far from discovering all protein families in nature.

## Author Summary

The rapidly emerging field of metagenomics seeks to examine the genomic content of communities of organisms to understand their roles and interactions in an ecosystem. Given the wide-ranging roles microbes play in many ecosystems, metagenomics studies of microbial communities will reveal insights into protein families and their evolution. Because most microbes will not grow in the laboratory using current cultivation techniques, scientists have turned to cultivation-independent techniques to study microbial diversity. One such technique—shotgun sequencing—allows random sampling of DNA sequences to examine the genomic material present in a microbial community. We used shotgun sequencing to examine microbial communities in water samples collected by the *Sorcerer II* Global Ocean Sampling (GOS) expedition. Our analysis predicted more than six million proteins in the GOS data—nearly twice the number of proteins present in current databases. These predictions add tremendous diversity to known protein families and cover nearly all known prokaryotic protein families. Some of the predicted proteins had no similarity to any currently known proteins and therefore represent new families. A higher than expected fraction of these novel families is predicted to be of viral origin. We also found that several protein domains that were previously thought to be kingdom specific have GOS examples in other kingdoms. Our analysis opens the door for a multitude of follow-up protein family analyses and indicates that we are a long way from sampling all the protein families that exist in nature.

## Abstract 3

The GOS data identified 6.12 million predicted proteins covering nearly all known prokaryotic protein families, and several new families. This almost doubles the number of known proteins and shows that we are far from identifying all the proteins in nature.

## Introduction

**Figure:**
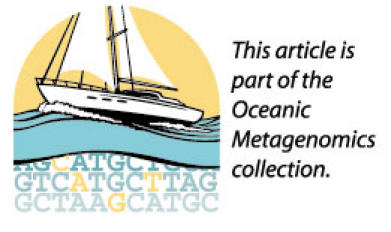


Despite many efforts to classify and organize proteins [1–6] from both structural and functional perspectives, we are far from a clear understanding of the size and diversity of the protein universe [7–9]. Environmental shotgun sequencing projects, in which genetic sequences are sampled from communities of microorganisms [10–14], are poised to make a dramatic impact on our understanding of proteins and protein families. These studies are not limited to culturable organisms, and there are no selection biases for protein classes or organisms. These studies typically provide a gene-centric (as opposed to an organism-centric) view of the environment and allow the examination of questions related to protein family evolution and diversity. The protein predictions from some of these studies are characterized both by their sheer number and diversity. For instance, the recent Sargasso Sea study [10] resulted in 1.2 million protein predictions and identified new subfamilies for several known protein families.

Protein exploration starts by clustering proteins into groups or *families* of evolutionarily related sequences. The notion of a protein family, while biologically very relevant, is hard to realize precisely in mathematical terms, thereby making the large-scale computational clustering and classification problem nontrivial. Techniques for these problems typically rely on *sequence similarity* to group sequences. Proteins can be grouped into families based on the highly conserved structural units, called *domains,* that they contain [15,16]. Alternatively, proteins are grouped into families based on their full sequence [17,18]. Many of these classifications, together with various expert-curated databases [19] such as Swiss-Prot [20], Pfam [15,21], and TIGRFAM [22,23], or integrated efforts such as Uniprot [24] and InterPro [25], provide rich resources for protein annotation. However, a vast number of protein predictions remain unclassified both in terms of structure and function. Given varying rates of evolution, there is unlikely to be a single similarity threshold or even a small set of thresholds that can be used to define every protein family in nature. Consequently, estimates of the number of families that exist in nature vary considerably based on the different thresholds used and assumptions made in the classification process [26–29].

In this study, we explored proteins using a comprehensive dataset of publicly available sequences together with environmental sequence data generated by the *Sorcerer II* Global Ocean Sampling (GOS) expedition [30]. We used a novel clustering technique based on full-length sequence similarity both to predict proteins and to group related sequences. The goals were to understand the rate of discovery of protein families with the increasing number of protein predictions, explore novel families, and assess the impact of the environmental sequences from the expedition on known proteins and protein families. We used hidden Markov model (HMM) profiling to examine the relative biases in protein domain distributions in the GOS data and existing protein databases. This profiling was also used to assess the impact of the GOS data on target selection for protein structure characterization efforts. We carried out in-depth analyses on several protein families to validate our clustering approach and to understand the diversity and evolutionary information that the GOS data added; the families included ultraviolet (UV) irradiation DNA damage repair enzymes, phosphatases, proteases, and the metabolic enzymes glutamine synthetase and RuBisCO.

## Results/Discussion

### Data Generation, Sequence Clustering, and HMM Profiling

We used the following publicly available datasets in this study (Table 1)—the National Center for Biotechnology Information (NCBI)'s nonredundant protein database (NCBI-nr) [31,32], NCBI Prokaryotic Genomes (PG) [31,33], TIGR Gene Indices (TGI-EST) [34], and Ensembl (ENS) [35,36]. The rationale for including these datasets is discussed in Materials and Methods. All datasets were downloaded on February 10, 2005.

**Table 1:**
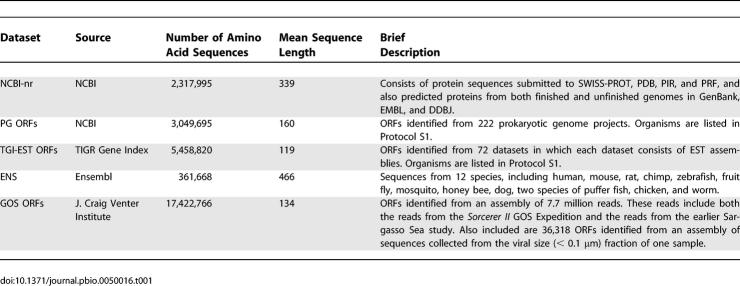
The Complete Dataset Consisted of Sequences from NCBI-nr, ENS, TGI-EST, PG, and GOS, for a Total of 28,610,944 Sequences

None of the above-mentioned databases contained sequences from the Sargasso Sea study [10], the largest environmental survey to date, and so we pooled reads from the Sargasso Sea study with the reads from the *Sorcerer II* GOS expedition [30], creating a combined set that we call the GOS dataset. The GOS dataset was assembled using the Celera Assembler [37] as described in [30] (see Materials and Methods). The GOS dataset was primarily generated from the 0.1 μm to 0.8 μm size filters and thus is expected to be mostly microbial [30]. The data also included a small set of sequences from a viral size (<0.1 μm) fraction (Table 1).

We identified open reading frames (ORFs) from the DNA sequences in the PG, TGI-EST, and GOS datasets. An ORF is commonly defined as a translated DNA sequence that begins with a start codon and ends with a stop codon. To accommodate partial DNA sequences, we extended this definition to allow an ORF to be bracketed by either a start codon or the start of the DNA sequence, and by either a stop codon or the end of the DNA sequence. ORFs were generated by considering translations of the DNA sequence in all six frames. For ORFs from the PG and TGI-EST datasets, we used the appropriate codon usage table for the known organism. For GOS ORFs from the assembled sequences, we used translation table 11 (the code for bacteria, archaea, and prokaryotic viruses) [31]. We did not include alternate codon translations in this analysis. For all datasets, only ORFs containing at least 60 amino acids (aa) were considered. Not all ORFs are proteins. In this paper, ORFs that have reasonable evidence for being proteins are called *predicted proteins;* other ORFs are called *spurious ORFs*.

In summary, the total input data for this study (Table 1) consisted of 28,610,994 sequences from NCBI-nr, PG, TGI-EST, ENS, and GOS. All data and analysis results will be made publicly available (see Materials and Methods).

We used a sequence similarity clustering to group related sequences and subsequently predicted proteins from this grouping. This approach of protein prediction was adopted for two reasons. First, the GOS data make up a major portion of the dataset being analyzed, and a large fraction of GOS ORFs are fragmentary sequences. Traditional annotation pipelines/gene finders, which presume complete or near-complete genomic data, perform unsatisfactorily on this type of data. Second, protein prediction based on the comparison of ORFs to known protein sequences imposes limits on the protein families that can be explored. In particular, novel proteins that belong to known families will not be detected if they are sufficiently distant from known members of that family. This is the case even though there may be other novel proteins that can transitively link them to the known proteins. Similarly, truly novel protein families will also not be detected.

As the primary input to our clustering process, we computed the pairwise sequence similarity of the 28.6 million aa sequences in our dataset using an all-against-all BLAST search [38]. This required more than 1 million CPU hours on two large compute clusters (see Materials and Methods). The sequences were clustered in four steps (see Materials and Methods). In the first step, we identified a nonredundant set of sequences from the entire dataset using only pairwise matches with ≥98% similarity and involving ≥95% of the length of the shorter sequence. This step served the dual role of identifying highly conserved groups of sequences (where each group was represented by a *nonredundant* sequence) and removing redundancy in the dataset due to identical and near-identical sequences. Only nonredundant sequences were considered for further steps in our clustering procedure. In the second step, we identified *core sets* of similar sequences using only matches between two sequences involving ≥80% of the length of the longer sequence. We used a graph-theoretic procedure to identify dense subgraphs (the core sets) within a graph defined by these matches. While the match parameters we used in this step were more relaxed than those in the first step, we chose them to reduce the grouping of unrelated sequences while simultaneously reducing the unnecessary splitting of families. In the third step, these core sets were transformed into profiles, and we used a profile–profile method [39] to merge related core sets into larger groups. In the final step, we recruited sequences to core sets using sequence-profile matching (PSI-BLAST [40]) and BLAST matches to core set members. We required the match to involve ≥60% of the length of the sequence being recruited.

We identified and removed clusters containing likely spurious ORFs using two filters (see Materials and Methods). The first filter identified clusters containing shadow ORFs. The second filter identified clusters containing conserved but noncoding sequences, as indicated by a lack of selection at the codon level. Only clusters that remained after the two filtering steps and contained at least two nonredundant sequences are reported in this analysis.

We examined the distribution of known protein domains in the full dataset using profile HMMs [41] from the Pfam [15] and TIGRFAM [22] databases (see Materials and Methods).

We labeled sequences that end up in clusters (containing at least two nonredundant sequences) or that have HMM matches as *predicted proteins.* The inclusion of the PG ORF set allowed for the evaluation of protein prediction using our clustering approach. A comparison of proteins predicted in the PG ORF set by our clustering against PG ORFs annotated as proteins by whole-genome annotation techniques revealed that our protein prediction method via clustering has a sensitivity of 83% and a specificity of 86% (see Materials and Methods). The HMM profiling allowed for the evaluation of our clustering technique's grouping of sequences. We used Pfam models in two different ways for this assessment (see Materials and Methods) and make three observations. First, using a simple Pfam domain architecture-based evaluation, these clusters are mostly consistent as reflected by 93% of clusters having less than 2% unrelated pairs of sequences in them. Second, these clusters are quite conservative and can split domain families, with 58% of domain architectures being confined to single clusters and 88% of domain architectures having more than half of their occurrences in a single cluster. Third, the size distribution of these clusters is quite similar to the size distribution of clusters induced by Pfams.

### Protein Prediction

Of the initial 28,610,944 sequences, we labeled 9,978,637 sequences (35%) as predicted proteins based on the clustering, of which nearly 60% are from GOS (Table 2). The HMM profiling labeled only an additional 226,743 (0.8%) sequences as predicted proteins, for a total of 10,205,380 predicted proteins. This indicates that our clustering method captures most of the sequences found by profile HMMs. For sequences both in clusters and with HMM matches, (on average) 73.5% of their length is covered by HMM matches. For sequences not in clusters but with HMM matches, this value is only 45.3%. Furthermore, while 64% of sequences in clusters have HMM matches, there are 3,550,901 sequences that are grouped into clusters but do not have HMM matches. Most of these clusters correspond either to families lacking profile HMMs or contain sequences that are too remote to match above the cutoffs used. The latter is an indication of the diversity added to known families that is not picked up by current profile HMMs.

**Table 2:**
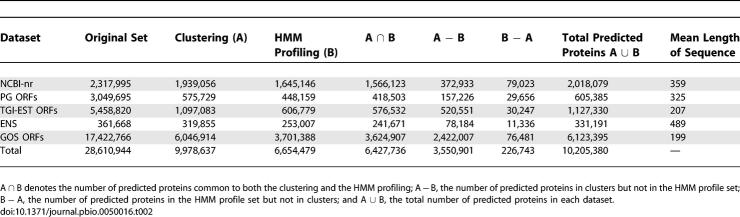
Clustering and HMM Profiling Results Showing the Number of Predicted Proteins (Including Both Redundant and Nonredundant Sequences) in Each Dataset

Using our method, the predicted proteins constitute different fractions of the totals for the five datasets, with 87% for NCBI-nr, nearly 20% for both PG ORFs and TGI-EST ORFs, 92% for ENS, and 35% for GOS. The high rate of prediction for ENS is a reflection of the high degree of conservation of proteins across the metazoan genomes, whereas the prediction rates for PG ORFs and TGI-EST ORFs are similar to rates seen in other protein prediction approaches. The 13% of NCBI-nr sequences that we marked as spurious may constitute contaminants in the form of false predictions or organism-specific proteins. Nearly two-thirds of these sequences are labeled “hypotheticals,” “unnamed,” or “unknown.” This is more than twice the fraction of similarly labeled sequences (30%) in the full NCBI-nr dataset. Of the remaining one-third, half of them are less than 100 aa in length. This suggests that they are either fast-evolving short peptides, spurious predictions, or proteins that failed to meet the length-based thresholds in the clustering.

Based on the clustering and the HMM profiling, there is evidence for 6,123,395 proteins in the GOS dataset (Table 2). Given the fragmentary nature of the GOS ORFs (as a result of the GOS assembly [10,30]), it is not surprising that the average length of a GOS-predicted protein (199 aa) is smaller than the average length of predicted proteins in NCBI-nr (359 aa), PG ORFs (325 aa), TGI-EST ORFs (207 aa), and ENS (489 aa). The ratio of clustered ORFs to total ORFs is significantly higher for the GOS ORFs (34%) compared to PG ORFs (19%). This could be due to a large number of false-positive protein predictions in the GOS dataset. However, this is unlikely for a variety of reasons. Nearly 4.64 million GOS ORFs (26.6%) have significant BLAST matches (with an *E-*value ≤1 × 10^−10^) to NCBI-nr sequences. The PG ORFs do not have a high false-positive rate compared to the submitted annotation for the prokaryotic genomes (see Materials and Methods). Most importantly, based on the fragmentary nature of GOS sequencing compared to PG sequencing, the number of shadow (spurious) ORFs ≥60 aa is significantly reduced (see Materials and Methods).

Some pairs of GOS-predicted proteins that belong to the same cluster are adjacent in the GOS assembly. While some of them correspond to tandem duplicate genes, an overwhelming fraction of the pairs are on mini-scaffolds [10], indicating that they are potentially pieces of the same protein (from the same clone) that we split into fragments. We estimate that this effect applies to 3% of GOS-predicted proteins. Sequencing errors and the use of the wrong translation table can also result in the ORF generation process producing split ORF fragments.

**Figure 1:**
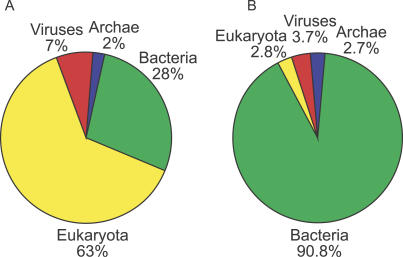
Proportion of Sequences for Each Kingdom (A) The combined set of NCBI-nr, PG, TGI-EST, and ENS has 3,167,979 sequences. The eukaryotes account for the largest portion and is more than twice the bacterial fraction. (B) Predicted kingdom proportion of sequences in GOS. Out of the 5,654,638 GOS sequences, 5,058,757 are assigned kingdoms using a BLAST-based scheme. The bacterial kingdom forms by far the largest fraction in the GOS set.

The combined set of predicted proteins in NCBI-nr, PG, TGI-EST, and ENS, as expected, has a lot of redundancy. For instance, most of the PG protein predictions are in NCBI-nr. Removing exact substrings of longer sequences (i.e., 100% identity) reduces this combined set to 3,167,979 predicted proteins. When we perform the same filtering on the GOS dataset, 5,654,638 predicted proteins remain. Thus, the GOS-predicted protein set is 1.8 times the size of the predicted protein set from current publicly available datasets. We used a simple BLAST based scheme to assign kingdoms for the GOS sequences (see Materials and Methods). Of the sequences that we could annotate by kingdom, 63% of the sequences in the public datasets are from the eukaryotic kingdom, and 90.8% of the sequences in the GOS set are from the bacterial kingdom (Figure 1).

### Protein Clustering

The 9,978,637 protein sequences predicted by our clustering method are grouped into 297,254 clusters of size two or more, where *size* of a cluster is defined to be the number of nonredundant sequences in the cluster. There are 280,187 small clusters (size < 20), 12,992 medium clusters (size between 20 and 200), and 4,075 large clusters (size > 200). While the 17,067 medium- and large-sized clusters constitute only 6% of the total number of clusters, they account for 85% of all the sequences that are clustered (Table 3). Many of the largest clusters correspond to families that have functionally diversified and expanded (Table 4). While some large families, such as the HIV envelope glycoprotein family and the immunoglobulins, also reflect biases in sequence databases, many more, including ABC transporters, kinases, and short-chain dehydrogenases, reflect their expected abundance in nature.

**Table 3:**
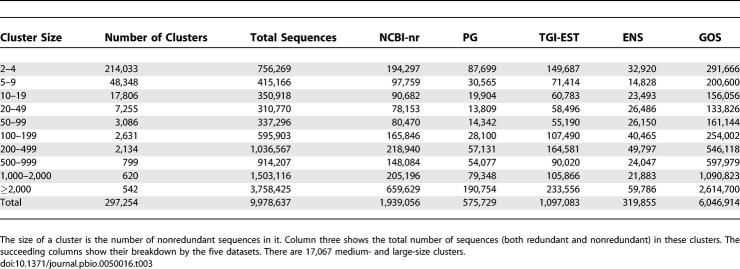
Cluster Size Distribution and the Distribution of Sequences in These Clusters

**Table 4:**
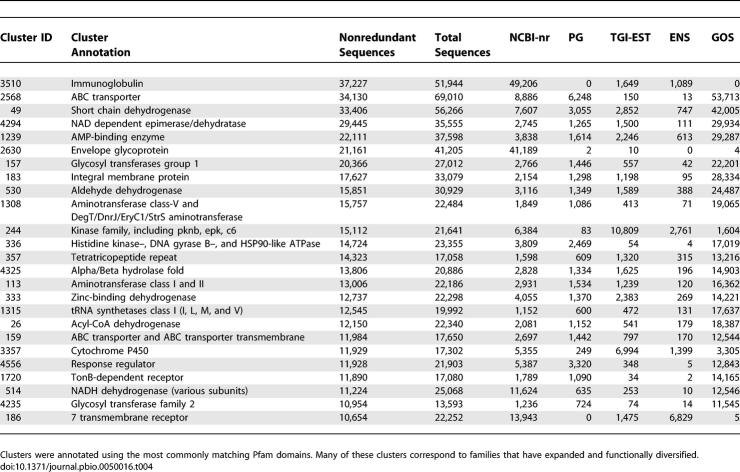
List of the Top 25 Clusters from the Clustering Process

### Rate of Discovery of Protein Families

**Figure 2:**
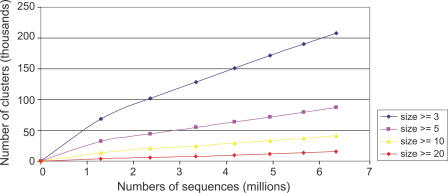
Rate of Discovery of Clusters as (Nonredundant) Sequences Are Added The *x*-axis denotes the number of sequences (in millions) and the *y*-axis denotes the number of clusters (in thousands). Seven datasets with increasing numbers of (nonredundant) sequences are chosen as described in the text. The blue curve shows the number of core sets of size ≥3 for the seven datasets. Curves for core set sizes ≥5, ≥10, and ≥20 are also shown. Linear regression gives slopes 0.027 (*R* ^2^ = 0.999), 0.011 (*R* ^2^ = 0.999), 0.0053 (*R* ^2^ = 0.999), and 0.0024 (*R* ^2^ = 0.996) for size ≥3, size ≥5, size ≥10, and size ≥20, respectively.

We examined the rate of discovery of protein families using our clustering method to determine whether our sampling of the protein universe is reaching saturation. We find that for the present number of sequences there is an approximately linear trend in the rate of discovery of clusters with the addition of new (i.e., nonredundant) sequences (Figure 2). Moreover, the observed distribution of cluster sizes is well approximated by a power law [42,43], and this observed power law can be used to predict the rate of growth of the number of clusters of a given size (see Materials and Methods). This rate is dependent on the value of the power law exponent and decreases with increasing cluster sizes. We find good agreement between the observed and predicted growth rates for different cluster sizes. The approximately linear relationship between the number of clusters and the number of protein sequences indicates that there are likely many more protein families (either novel or subfamilies distantly related to known families) remaining to be discovered.

### GOS versus Known Prokaryotic versus Known Nonprokaryotic

**Figure 3:**
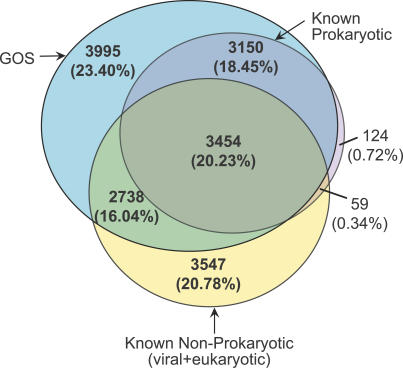
Venn Diagram Showing Breakdown of the 17,067 Medium and Large Clusters by Three Categories—GOS, Known Prokaryotic, and Known Nonprokaryotic

We also examined the GOS coverage of known proteins and protein families. Based on the cell-size filtering performed while collecting the GOS samples, we expected that the sample would predominantly be a size-limited subset of prokaryotic organisms [30]. We studied the content of the 17,067 medium- and large-sized clusters across three groupings: (1) GOS, (2) known prokaryotic (PG together with bacterial and archaeal portions of NCBI-nr), and (3) known nonprokaryotic (TGI-EST and ENS together with viral and eukaryotic portions of NCBI-nr). The Venn diagram in Figure 3 shows the breakdown of these clusters by content (see Materials and Methods). The largest section contains GOS-only clusters (23.40%) emphasizing the significant novelty provided by the GOS data. The next section consists of clusters containing sequences from only the known nonprokaryotic grouping (20.78%), followed closely by the section containing clusters with sequences from all three groupings (20.23%). The large known nonprokaryotic–only grouping shows that our current GOS sampling methodology will not cover all protein families, and perhaps misses some protein families that are exclusive to higher eukaryotes. The large section of clusters that include all three groupings indicates a large core of well-conserved protein families across all domains of life. In contrast, the known prokaryotic protein families are almost entirely covered by the GOS data.

### Novelty Added by GOS Data

There are 3,995 medium and large clusters that contain only sequences from the GOS dataset. Some are divergent members of known families that failed to be merged by the clustering parameters used, or are too divergent to be detected by any current homology detection methods. The remaining clusters are completely novel families. In exploring the 3,995 GOS-only clusters, 44.9% of them contain sequences that have HMM matches, or BLAST matches to sequences in a more recent snapshot of NCBI-nr (downloaded in August 2005) than was used in this study. The recent NCBI-nr matches include phage sequences from cyanophages (P-SSM2 and P-SSM4) [44] and sequences from the SAR-11 genome (*Candidatus pelagibacter ubique* HTCC1062) [45]. We used profile–profile searches [39] to show that an additional 12.5% of the GOS-only clusters can be linked to profiles built from Protein Data Bank (PDB), COG, or Pfam. The 2,295 clusters with detected homology are referred to as Group I clusters. The remaining 1,700 (42.6%) GOS-only clusters with no detectable homology to known families are labeled as Group II clusters.

We applied a guilt-by-association operon method to annotate the GOS-only clusters with a strategy that did not rely on direct sequence homology to known families. Function was inferred for the GOS-only clusters by examining their same-strand neighbors on the assembly (see Materials and Methods). Similar strategies have been successfully used to infer protein function in finished microbial genomes [46–48]. Despite minimal assembly of GOS reads, many scaffolds and mini-scaffolds contain at least partial fragments of more than one predicted ORF, thereby making this approach feasible. For 90 (5.3%) of the Group II clusters, and for 214 (9.3%) of the Group I clusters, at least one Gene Ontology (GO) [49] biological process term at *p*-value ≤0.05 can be inferred. The inferred functions and neighbors of some of these GOS-only clusters are highlighted in Table 5. We observed that for Group I clusters, the neighbor-inferred function is often bolstered by some information from weak homology to known sequences. While neighboring clusters as a whole are of diverse function, a number of GOS-only clusters seem to be next to clusters implicated in photosynthesis or electron transport. These GOS-only clusters could be of viral origin, as cyanophage genomes contain and express some photosynthetic genes that appear to be derived from their hosts [44,50,51]. In support of these observations, we identified five photosynthesis-related clusters containing hundreds to thousands of viral sequences, including psbA, psbD, petE, SpeD, and hli in the GOS data; furthermore, our nearest-neighbor analysis of these sequences reveals the presence of multiple viral proteins (unpublished data).

**Table 5:**
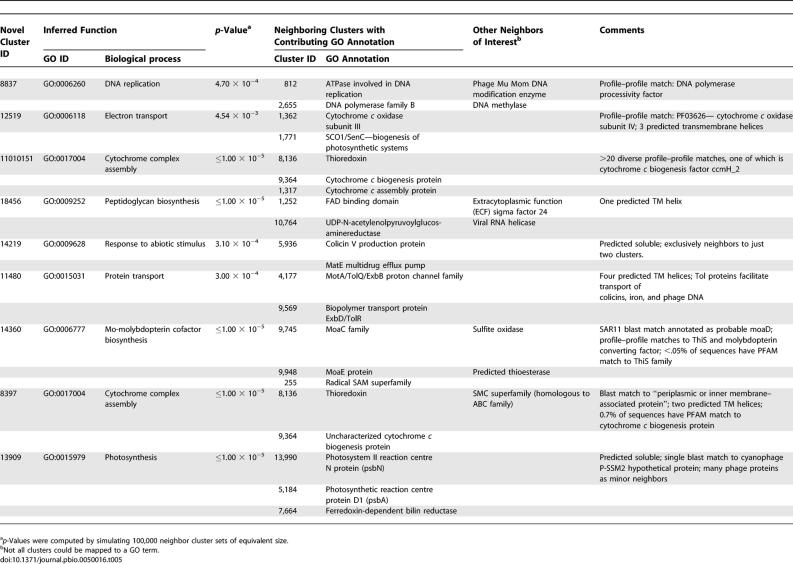
Neighbor-Based Inference of Function for Novel Clusters of GOS Sequences

**Figure 4:**
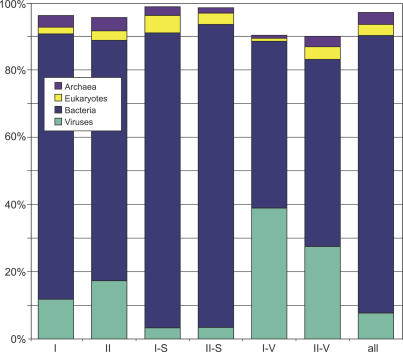
Enrichment in the GOS-Only Set of Clusters for Viral Neighbors Cluster sets from left to right are: I, GOS-only clusters with detectable BLAST, HMM, or profile-profile homology (Group I); II, GOS-only clusters with no detectable homology (Group II); I-S, a sample from all clusters chosen to have the same size distribution as Group I; II-S, a sample from all clusters chosen to have the same size distribution as Group II; I-V, a subset of clusters in Group I containing sequences collected from the viral size fraction; II-V, a subset of clusters in Group II from the viral size fraction; and all clusters. Notice that although predominantly bacterial, GOS-only clusters are assigned as viral based on their neighbors more often than the size-matched samples and the set of all clusters.

Although the majority of GOS-only sequences are bacterial, a higher than expected proportion of the GOS-only clusters are predicted to be of viral origin, implying that viral sequences and families are poorly explored relative to other microbes. To assign a kingdom to the GOS-only clusters, we first inferred the kingdom of neighboring sequences based on the taxonomy of the top four BLAST matches to the NCBI-nr database (see Materials and Methods). A possible kingdom was assigned to the GOS-only cluster if more than 50% of assignable neighboring sequences belong to the same kingdom. Viewed in this way, 11.8% of Group I clusters and 17.3% of Group II clusters with at least one kingdom-assigned neighbor have more than 50% viral neighbors (Figure 4). Only 3.3% and 3.4% of random samples of clusters with size distributions matching that of Group I and Group II clusters have more than 50% viral neighbors, while 7.7% of all clusters pass this criterion. A total of 547 GOS-only clusters contain sequences collected from the viral size fraction included in the GOS dataset. For these clusters, 38.9% of the Group I subset and 27.5% of the Group II subset with one or more kingdom-assigned neighbors would be inferred as viral, based on the conservative criteria of having more than 50% viral assignable neighbors. Several alternative kingdom assignment methods were tried (see Materials and Methods) and provide for a similar conclusion.

The GOS-only clusters also tend to be more AT-rich than sequences from a random size-matched sample of clusters (35.9% ± 8% GC content for Group II clusters versus 49.5% ± 11% GC content for sample). Phage genomes with a *Prochlorococcus* host [44] are also AT rich (37% average GC content). Our analysis of the graph constructed based on inferred operon linkages between all clusters indicates that the GOS-only clusters may constitute large sets of cotranscribed genes (see Materials and Methods).

The high proportion of potentially viral novel clusters observed here is reasonable, as 60%–80% of the ORFs in most finished marine phage genomes are not homologous to known protein sequences [52]. Viral metagenomics projects have reported an equally high fraction of novel ORFs [53], and a recent marine metagenomics project estimated that up to 21% of photic zone sequences could be of viral origin [51]. It has also been reported that 40% of ORFans (sequences that lack similarity to known proteins and predicted proteins) exist in close spatial proximity to each other in bacterial genomes, and this combined with proximity to integration signals has been used to suggest a viral horizontally transferred origin for many bacterial ORFans [54]. Others have noted a clustering of ORFans in genome islands and suggested they derive from a phage-related gene pool [55]. A recent analysis of genome islands from related *Prochlorococcus* found that phage-like genes and novel genes cohabit these dynamic areas of the genome [56]. In our GOS-only clusters, 37 of the 1,700 clusters with no detectable similarity (2.2%) have at least ten bacterial-classified and ten viral-classified neighboring ORFs. This is 6.2-fold higher than the rate seen for the size-matched sample of all clusters (six clusters, 0.35%). This would seem to add more support to a phage origin for at least some ORFans found in bacterial genomes.

If a sizable portion of the novel families in the GOS data are in fact of viral origin, it suggests that we are far from fully exploring the molecular diversity of viruses, a conclusion echoed in previous studies of viral metagenomes [53,57,58]. In studies of bacterial genomes, discovery of new ORFans shows no sign of reaching saturation [59]. Coverage of many phage families in the GOS data may be low, given that there are inherent differences in the abundance of their presumed bacterial hosts. These GOS-only clusters were operationally defined as having at least 20 nonredundant sequences. Reducing this threshold to ten nonredundant sequences adds 7,241 additional clusters. Whether this vast diversity represents new families or is a reflection of the inability to detect distant homology will require structural and biochemical studies, as well as continued development of computational methods to identify remotely related sequences.

### Comparison of Domain Profiles in GOS and PG Datasets

We used HMM profiling to address the question of which biochemical and biological functions are expanded or contracted in GOS compared to the largely terrestrial genomes in PG. Significant differences are seen in 68% of domains (4,722 out of the 6,975 domains that match either GOS or PG; *p*-value <0.001, chi-square test). These differences reflect several factors, including differing biochemical needs of oceanic life and taxonomic biases in the two datasets. An initial comparison of these domain profiles helps shed light on these factors. 91% (964/1,056) of GOS-only domains are viral and/or eukaryotic specific (by Pfam annotation). Most of the remaining 92 domains are rare (63 domains have less than ten copies in GOS), are predominantly eukaryotic/viral, or are specific to narrow bacterial taxa without completed genome sequences. Most of the 879 PG-only domains are also rare (444 have ten or less members), and/or are restricted to tight lineages, such as *Mycoplasma* (104 matches to five domains) or largely extremeophile archaeal-specific domains (1,254 matches to 99 domains). Highly PG-enriched domains also tend to belong in these categories. Many moderately skewed domains reflect the taxonomic skew between PG and GOS. For instance, we found that a set of six sarcosine oxidase-related domains are 4.8-fold enriched in GOS (Table 6). They are mostly found in α- and γ-proteobacteria, which are widespread in GOS. Normalizing to the taxonomic class level predicts a 1.8-fold enrichment in GOS, indicating that taxonomy alone cannot fully explain the prevalence of these proteins in oceanic bacteria.

**Table 6:**
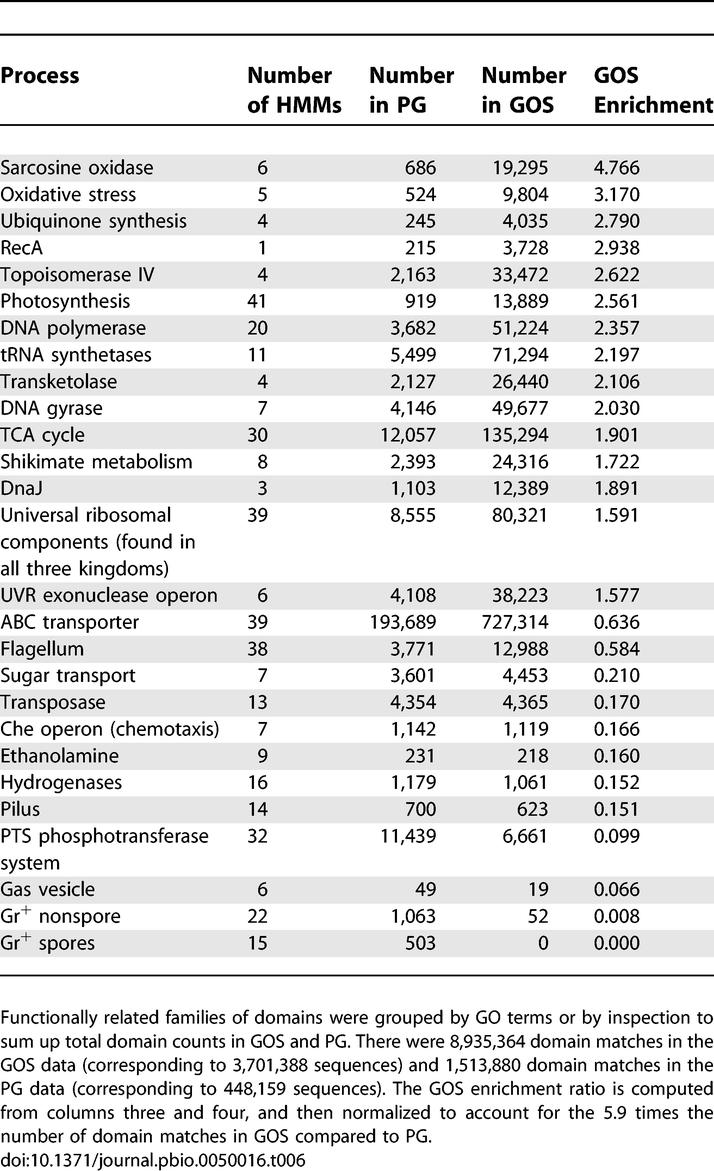
Functions Skewed in Domain Representation between PG and GOS

### Mysterious Lack of Characteristic Gram-Positive Domains

Gram-positive bacteria *(Firmicutes* and *Actinobacteria)* represent 26.7% of PG and ~12% of GOS [30]. Given the larger size of the GOS dataset, one might predict Gram-positive–specific domains to be ~2.4-fold enriched in GOS. Instead, the opposite is consistently seen. Of 15 firmicute-specific spore-associated domains, PG has 503 members, but GOS has none. For another 22 firmicute-restricted domains of varying or unknown function, the PG/GOS ratio is 1797:77 (Table 6). Hence, it appears that GOS Gram-positive lineages lack most of their characteristic protein domains. Two sequenced marine Gram-positives (Oceanobacillus iheyensis [60] and Bacillus sp. NRRL B-14911) have a large complement of these domains. However, another recently assembled genome from Sargasso sea surface waters, the actinomycete Janibacter sp. HTCC2649, has just two of these domains, and may reveal a whole-genome context for this curious loss of characteristic domains.

### Flagellae and Pili Are Selectively Lost from Oceanic Species

Flagellum components from both eubacteria and archaea are significantly underrepresented in the GOS dataset by about 2-fold (Table 6). Ironically, at a bacterial scale, swimming may be worthwhile on an almost dry surface, but not in open water. The chemotaxis (che) operon that often directs flagellar activity is also rare in GOS. Another directional appendage, the pilus, is even more reduced, though its taxonomic distribution (mostly in proteobacteria, predominantly γ-proteobacteria) would have predicted enrichment.

### Skew in Core Cellular Pathways

While taxonomically specialized domains are likely to be skewed by taxonomic differences, core pathways found in many or all organisms paint a different picture. We used GO term mapping and text mining to group domains into major functions and to look for consistent skews across several domains. Several core functions, including DNA-associated proteins (DNA polymerase, gyrase, topoisomerase), ribosomal subunits shared by all three kingdoms, marker proteins such as recA and dnaJ, and TCA cycle enzymes all tend to be GOS enriched. This suggests that oceanic genomes may be more compact than sequenced genomes and so have a higher proportion of core pathways.

### Characteristics and Kingdom Distribution of Known Protein Domains

A decade ago, databases were highly biased towards proteins of known function. Today, whole-genome sequencing and structural genomics efforts have presumably reduced the biases that are a result of targeted protein sequencing. We used the Pfam database to compare the characteristics and kingdom distribution of known protein domains in the GOS dataset to that of proteins in the publicly available datasets (NCBI-nr, PG, TGI-EST, and ENS). Such an effort can be used to assess biases in these datasets, help direct future sampling efforts (of underrepresented organisms, proteins, and protein families), make more informed generalizations about the protein universe, and provide important context for determination of protein evolutionary relationships (as biased sampling could indicate expected but missing sequences).

For this analysis we used the nonredundant datasets (at 100% identity) discussed in Figure 1. We refer to the set of 3,167,979 nonredundant sequences from NCBI-nr, PG, TGI-EST, and ENS as the public-100 set and the similarly filtered set of 5,654,638 sequences from the GOS data as the GOS-100 set*.*

**Figure 5:**
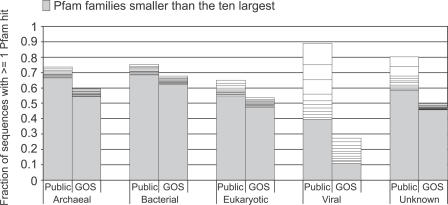
Coverage of GOS-100 and Public-100 by Pfam and Relative Sizes of Pfam Families by Kingdom, Sorted by Size The public-100 sequences are annotated using the NCBI taxonomy and the source public database annotations. GOS-100 sequences were given kingdom weights as described in Materials and Methods. For each kingdom, the fraction of sequences with ≥1 Pfam match are shown, while the ten largest Pfam families shown as discrete sections whose size is proportional to the number of matches between that family and GOS-100 or public-100 sequences. Pfam families that are smaller than the ten largest are binned together in each column's bottom section. Pfam covers public-100 better than GOS-100 in all kingdoms, with the greatest difference occurring in the viral kingdom, where 89.1% of public-100 viral sequences match a Pfam domain, while only 27.5% of GOS-100s have a sequence match.

About 70% of public-100 sequences and 56% of GOS-100 sequences significantly match at least one Pfam model. The most obvious difference between the sets is that the vast majority of GOS sequences are bacterial, and this has to be taken into account when comparing the numbers. Since different Pfam families appear with different frequencies in the kingdoms, we considered the results for each kingdom separately (Figure 5). We then evaluated all kingdoms together, with results normalized by relative abundance of members from the different kingdoms. A domain found commonly and exclusively in eukaryotes and abundant in public-100 would be expected to be found rarely in GOS-100. We used a conservative BLAST-based kingdom assignment method to assign kingdoms to the GOS sequences (see Materials and Methods).

In each kingdom, sequences in GOS-100 are less likely to match a Pfam family than those in public-100 (Figure 5). For the cellular kingdoms, these differences are comparatively modest. While diversity of the GOS data accounts for some of this difference, it might also be explained in part by the fragmentary nature of the GOS sequences. Viruses tell a dramatic and different story. Of public-100 viral sequences, 89.1% match a Pfam domain, while only 27.5% of GOS-100 viral sequences have a match. This tremendous difference appears to be due to heavy enrichment of the public data for minor variants of a few protein families, indicated by the sizes of the ten most populous Pfams in each kingdom (Figure 5). Sequences from three Pfam families (envelope glycoprotein GP120, reverse transcriptase, and retroviral aspartyl protease) account for a third of all public viral sequences. By contrast, the most populous three families in the GOS-100 data (bacteriophage T4-like capsid assembly protein [Gp20], major capsid protein Gp23, and phage tail sheath protein) account for only about 7% of public-100 sequences. Such a difference may be due to intentional oversampling of proteins that come from disease-causing organisms in the public dataset.

While the total proportion of proteins with a Pfam hit is fairly similar between public-100 (70%) and GOS-100 (56%) datasets, there are considerable differences with regard to the distributions of protein families within these two datasets. The most highly represented Pfam families in GOS-100 compared to public-100 are shown in Table 7. Notably, we found that while many known viral families are absent in GOS-100, viral protein families dominate the list of the families more highly represented in GOS-100; this is presumably because of biases in the collection of previously known viral sequences. Surprisingly few bacterial families were among the most represented in GOS-100 compared with public-100. By contrast, we also observed that those families found more rarely in GOS-100 than public-100 were frequently bacterial (Table 7). This appears to be a result of the large number of key bacterial and viral pathogen proteins in public-100 that are comparatively less abundant in the oceanic samples and/or less intensively sampled.

**Table 7:**
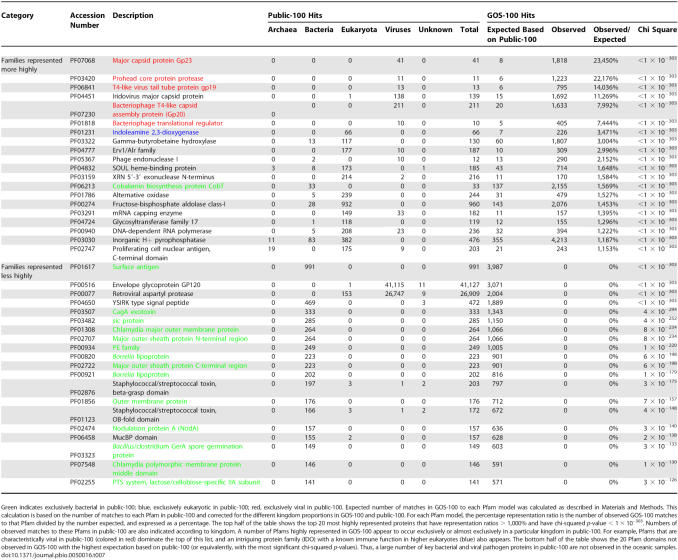
Top Pfam Families Represented More Highly or Less Highly in GOS-100 than in Public-100

### GOS-100 Data Suggest That a Number of “Kingdom-Specific” Pfams Actually Are Represented in Multiple Kingdoms

Of the 7,868 Pfam models in Pfam 17.0, 4,050 match proteins from only a single kingdom in public-100. The additional sequences from GOS-100 reveal that some of these families actually have representatives in multiple kingdoms. Table 8 shows 12 families that have a Pfam match to at least one GOS-100 protein with an *E-*value ≤ 1 × 10^−10^, and which we confidently assigned to a kingdom different from that of all the public-100 matches. Because our criteria for a “confident” kingdom assignment are conservative, there are only one or a few confident assignments for each Pfam domain to a “new” kingdom. Our “confident” criteria are especially difficult to meet in the case of kingdom-crossing, due to the votes contributed by the crossing protein (see Materials and Methods). Thus, many scaffolds have no confident kingdom assignment. Our examination of each of the scaffolds responsible for a determination of kingdom-crossing confirms that each one had both a highly significant match to the Pfam model in question and an overwhelming number of votes for the unexpected kingdom. These scaffold assemblies were also manually inspected. No clear anomalies were observed. In most instances, the assemblies in question were composed of a single unitig, and as such are high-confidence assemblies. Mate pair coverage and consistent depth of coverage provide further support for the correctness of those assemblies that are built from multiple unitigs. Examples of kingdom-crossing families include indoleamine 2,3-dioxygenase (IDO), MAM domain, and MYND finger [15], which have previously only been seen in eukaryotes, but we find them also to be present in bacteria. These Pfams now cross kingdoms, due either to their being more ancient than previously realized or to lateral transfer.

**Table 8:**
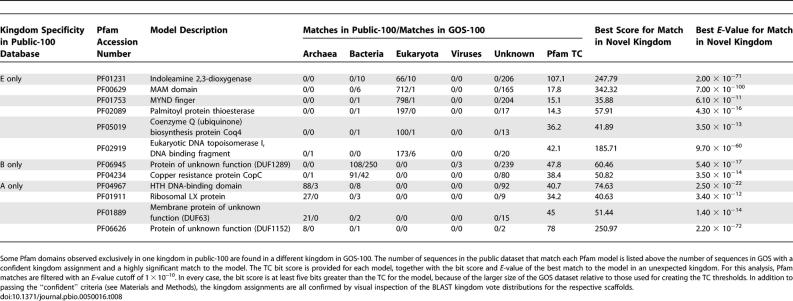
New Multi-Kingdom Pfams

**Figure 6:**
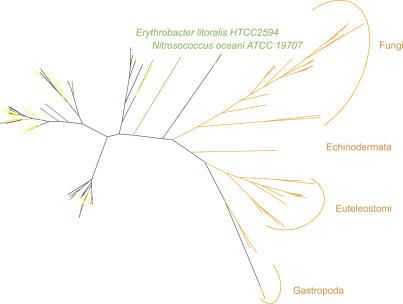
Maximum Likelihood Phylogeny for the IDO Family The phylogeny is based on an alignment of 93 sequences from GOS-100 and 51 sequences from public-100 and NCBI-nr from March 2006 that matched the IDO Pfam model and satisfied multiple alignment quality criteria. The IDO family is eukaryotic specific in public-100. The phylogeny shows a clade with all the GOS sequences, predicted to be bacterial (navy blue), eukaryotic (yellow), or unknown (gray), along with two sequences from the marine bacteria Erythrobacter litoralis and *Nitrosococcus oceani* (lime green) submitted to the sequence database after February 2005, and a public-only clade of only eukaryotic sequences (orange).

We explored the IDO family further. This family has representatives in vertebrates, invertebrates, and multiple fungal lineages [15,61] in public-100. Members of the IDO family are heme-binding, and mammalian IDOs catalyze the rate-limiting step in the catabolic breakdown of tryptophan [62], while family members in mollusks have a myoglobin function [63]. In mammals, IDO also appears to have a role in the immune system [62,64–66]. The IDO Pfam has matches to 66 proteins in public-100, all of which are eukaryotic. However, it also has matches to ten GOS-100 sequences that we confidently labeled as bacterial proteins and matches to 206 GOS-100 sequences for which a confident kingdom assignment could not be made (many of these are likely bacterial sequences due to the GOS sampling bias). To reconstruct a phylogeny of the IDO family, we searched a recent version of NCBI-nr (March 5, 2006) for IDO proteins that were not included in the public-100 dataset. The search identified two bacterial proteins from the whole genomes of the marine bacteria Erythrobacter litoralis and *Nitrosococcus oceani,* and 24 eukaryotic proteins (see Materials and Methods). The phylogeny shown in Figure 6 shows 54% bootstrap support for a separation of the clade containing exclusively public-100 and NCBI-nr 2006 eukaryotic sequences from a clade with the GOS-100 sequences as well as the two NCBI-nr E. litoralis and N. oceani sequences. We confirmed this feature of the tree topology with multiple other phylogeny reconstruction methods. Curiously, there is considerable intermixing of bacterial and eukaryotic sequences in the clade of GOS-100 sequences and the two NCBI-nr bacteria. A manual inspection of the scaffolds that contain the ten GOS-100 sequences (containing the IDO domain) that we confidently labeled as bacterial, overwhelmingly supports the kingdom assignment. However, a manual inspection of the scaffolds that contain the ten GOS-100 sequences (containing the IDO domain) that we confidently labeled as eukaryotes presents a less convincing picture. These scaffolds are short, with most of them containing only two voting ORFs. Since the NCBI-nr version used in the public-100 set has IDO from eukaryotes only, the ORF with the IDO domain itself would cast four votes for eukaryotes. Thus, these GOS-100 eukaryotic labelings are not nearly as confident as the ones labeled bacterial.

### Structural Genomics Implications

Knowledge about global protein distributions can be used to inform priorities in related fields such as structural genomics. Structural genomics is an international effort to determine the 3-D shapes of all important biological macromolecules, with a primary focus on proteins [67–72]. Previous studies have shown that an efficient strategy for covering the protein structure universe is to choose protein targets for experimental structure characterization from among the largest families with unknown structure [73,74]. If the structure of one family member is determined, it may be used to accurately infer the fold of other family members, even if the sequence similarity between family members is too low to enable accurate structural modeling [75]. Therefore, large families are a focus of the production phase of the Protein Structure Initiative (PSI), the National Institutes of Health–funded structural genomics project that commenced in October 2005 [76].

In March 2005, 2,729 (36%) of 7,677 Pfam families had at least one member of known structure; these families could be used to infer folds for approximately 51% of all pre-GOS prokaryotic proteins (covering 44% of residues) [74]. The Pfam5000 strategy is to solve one structure from each of the largest remaining families, until a total of 5,000 families have at least one member with known structure [73]. As this strategy is similar to that being used at PSI centers to choose targets, projections based on the Pfam5000 should reflect PSI results. Completion of the Pfam5000, a tractable goal within the production phase of PSI, would enable accurate fold assignment for approximately 65% of all pre-GOS prokaryotic proteins. In the GOS-100 dataset, we observed that 46% of the proteins might currently be assigned a fold based on Pfam families of known structure (see Materials and Methods). Completion of the Pfam5000 would increase this coverage to 55%.

The GOS sequences will affect Pfam in two ways: some will be classified in existing protein families, thus increasing the size of these families; others may eventually be classified into new GOS-specific families. Both of these will alter the relative sizes of different families, and thus their prioritization for structural genomics studies. We calculated the sizes for all Pfam families based on the number of occurrences of each family in the public-100 dataset. Proteins in GOS-100 were then added and the family sizes were recalculated. A total of 190 families that are not in the Pfam5000 based on public-100 are moved into the Pfam5000 after addition of the GOS data. The 30 largest such families are shown in Table 9. As 20 of the 30 families are annotated as domains of unknown function in Pfam, structural characterization might be helpful in identifying their cellular or molecular functions. Reshuffling the Pfam5000 to prioritize these 190 families would improve structural coverage of GOS sequences after completion of the Pfam5000 by almost 1% relative to the original Pfam5000 (from 55.4% to 56.1%), with only a small decrease in coverage of public-100 sequences (from 67.7% to 67.5%).

**Table 9:**
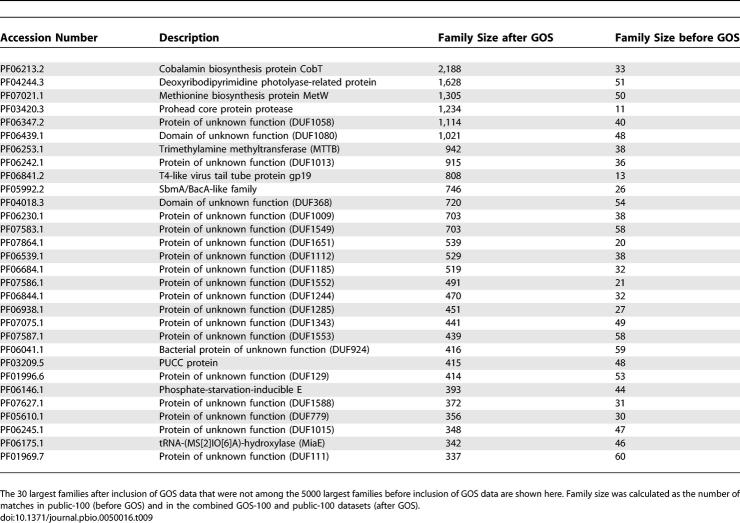
The 30 Largest Structural Genomics Target Families Added to the Pfam5000 Based on Inclusion of GOS Sequences

The Pfam5000 would be further reprioritized by the classification of clusters of GOS sequences into Pfam. Assuming each cluster of pooled GOS-100 and public-100 sequences without a current Pfam match would be classified as a single Pfam family, 885 such families would replace existing families in the Pfam5000. These 885 clusters contain a total of 383,019 proteins in GOS-100 and public-100. The reprioritized Pfam5000 would also retain 1,183 families of unknown structure from the current Pfam5000; these families comprise a total of 1,040,330 proteins in GOS-100 and public-100.

### Known Protein Families and Increased Diversity Due to GOS Data

Several protein families serve as examples to further highlight the diversity added by the GOS dataset. In this paper, we examined UV irradiation DNA damage repair enzymes, phosphatases, proteases, and the metabolic enzymes glutamine synthetase and RuBisCO (Table 10). The RecA family (unpublished data) and the kinase family [77] have also been explored in the context of the GOS data. There are more than 5,000 RecA and RecA-like sequences in the GOS dataset (Table 10). An analysis of the RecA phylogeny including the GOS data reveals several completely new RecA subfamilies. A detailed study of kinases in the GOS dataset demonstrated the power of additional sequence diversity in defining and exploring protein families [77]. The discovery of 16,248 GOS protein kinase–like enzymes enabled the definition and analysis of 20 distinct kinase-like families. The diverse sequences allowed the definition of key residues for each family, revealing novel core motifs within the entire superfamily, and predicted structural adaptations in individual families. This data enabled the fusion of choline and aminoglycoside kinases into a single family, whose sequence diversity is now seen to be at least as great as the eukaryotic protein kinases themselves.

**Table 10:**
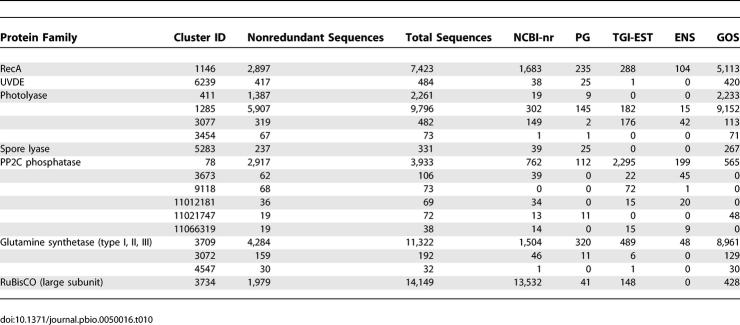
Clustering of Sequences in Families That Are Explored in This and Companion Papers

### Proteins Involved in the Repair of UV-Induced DNA Damage

Much of the attention in studies of the microbes in the world's oceans has justifiably focused on phototrophy, such as that carried out by the proteorhodopsin proteins. Previously, in the Sargasso Sea study [10] it was shown that shotgun sequencing reveals a much greater diversity of proteorhodopsin-like proteins than was previously known from cloning and PCR studies. However, along with the potential benefits of phototrophy come many risks, such as the damage caused to cells by exposure to solar irradiation, especially the UV wavelengths. Organisms deal with the potential damage from UV irradiation in several ways, including protection (e.g., UV absorption), tolerance, and repair [78]. Our examination of the protein family clusters reveals that the GOS data provides an order of magnitude increase in the diversity (in both numbers and types) of homologs of proteins known to be involved in pathways specifically for repairing UV damage.

One aspect of the diversity of UV repair genes is seen in the overrepresentation of photolyase homologs in the GOS data (see Table 10). Photolyases are enzymes that chemically reverse the UV-generated inappropriate covalent bonds in cyclobutane pyrimidine dimers and 6–4 photoproducts [79]. The massive numbers of homologs of these proteins in the GOS data (11,569 GOS proteins in four clusters; see Table 10) is likely a reflection of their presence in diverse species and the existence of novel functions in this family. New repair functions could include repair of other forms of UV dimers (e.g., involving altered bases), use of novel wavelengths of light to provide the energy for repair, repair of RNA, or repair in different sequence contexts. In addition, some of these proteins may be involved in regulating circadian rhythms, as seen for photolyase homologs in various species. Our findings are consistent with the recent results of a comparative metagenomic survey of microbes from different depths that found an overabundance of photolyase-like proteins at the surface [51].

**Figure 7:**
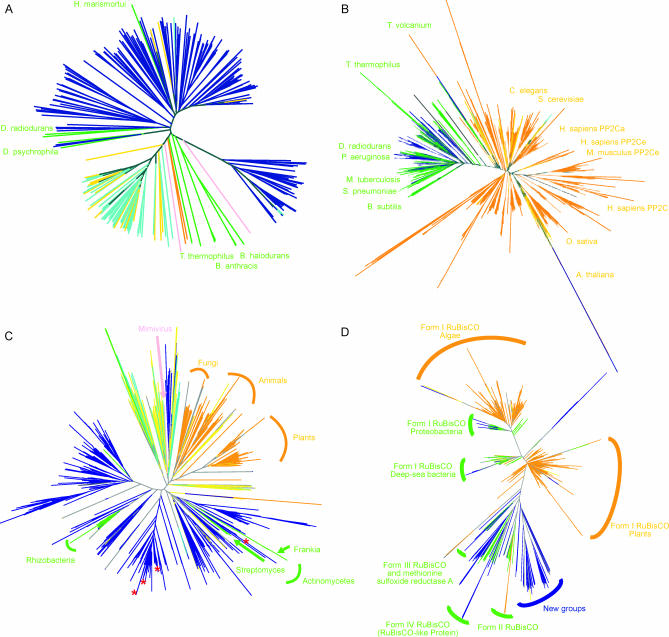
Phylogenies Illustrating the Diversity Added by GOS Data to Known Families That We Examined Kingdom assignments of the sequences are indicated by color: yellow, GOS-eukaryotic; navy blue, GOS-bacterial/archaeal; aqua, GOS-viral; orange, NCBI-nr–eukaryotic; lime green, NCBI-nr–bacterial/archaeal; pink, NCBI-nr–viral; gray, unclassified. (A) Phylogeny of UVDE homologs. (B) Phylogeny of PP2C-like sequences. (C) Phylogeny of type II GS gene family. In addition to the large amount of diversity of bacterial type II GS in the GOS data, a large group of GOS viral sequences and eukaryotic GS co-occur at the top of the tree with the eukaryotic virus Acanthamoeba polyphaga mimivirus (shown in pink). The red stars indicate the locations of eight type II GS sequences found in the type I–type II GS gene pairs. They are located in different branches of the phylogenetic tree. The rest of the type II GS sequences were filtered out by the 98% identity cutoff. (D) Phylogeny of the homologs of RuBisCO large subunit. A large portion of the RuBisCO sequences from the GOS data forms new branches that are distinct from the previously known RuBisCO sequences in the NCBI-nr database.

A good deal was known about the functions and diversity of photolyases prior to this project. However, much less is known about other UV damage–specific repair enzymes, and examination of the GOS data reveals a remarkable diversity of each of these. For example, prior to this project, there were only some 25 homologs of UV dimer endonucleases (UVDEs) available [80], and most of these were from the *Bacillus* species. There are 420 homologs of UVDE (cluster 6239) in the GOS data representing many new subfamilies (Figure 7A and Materials and Methods). A similar pattern is seen for spore lyases (which repair a UV lesion specific to spores [81]) and the pyrimidine dimer endonuclease (DenV, which was originally identified in T4 phage [82]). We believe this will also be true for UV dimer glycosylases [83], but predictions of function for homologs of these genes are difficult since they are in a large superfamily of glycosylases.

Our analysis of the kingdom classification assignments suggests that the diversity of UV-specific repair pathways is seen for all types of organisms in the GOS samples. This apparently extends even to the viral world (e.g., 51 of the UVDE homologs are assigned putatively to viruses), suggesting that UV damage repair may be a critical function that phages provide for themselves and their hosts in ocean surface environments. Based on the sheer numbers of genes, their sequence diversity, and the diversity of types of organisms in which they are apparently found, we conclude that many novel UV damage–repair processes remain to be discovered in organisms from the ocean surface water.

### Evidence of Reversible Phosphorylation in the Oceans

Reversible phosphorylation of proteins represents a major mechanism for cellular processes, including signal transduction, development, and cell division [84]. The activity of protein kinases and phosphatases serve as antagonistic regulators of the cellular response. Protein phosphatases are divided into three major groups based on substrate specificity [85]. The Mg^2+^- or Mn^2+^-dependent phosphoserine/phosphothreonine protein phosphatase family, exemplified by the human protein phosphatase 2C (PP2C), represents the smallest group in number. An understanding of their physiological roles has only recently begun to emerge. In eukaryotes, one of the major roles of PP2C activity is to reverse stress-induced kinase cascades [86–89].

We identified 613 PP2C-like sequences in the GOS dataset, and they are grouped into two clusters (Table 10). These sequences contain at least seven motifs known to be important for phosphatase structure and function [90,91]. Invariant residues involved in metal binding (aspartate in motifs I, II, VIII) and phosphate ion binding (arginine in motif I) are highly conserved among the GOS sequences.

Using the catalytic domain portion of these sequences we constructed a phylogeny showing that despite the overall conserved structure of the PP2C family of proteins, the known bacterial PP2C-like sequences group together with the GOS bacterial PP2C-like sequences (Figure 7B, Materials and Methods). Furthermore, the eukaryotic PP2Cs display a much greater degree of sequence divergence compared to the bacterial PP2C sequences.

We also examined the combined dataset of PP2C-like phosphatases further for potential differences in amino acid composition between the bacterial and eukaryotic groups. We observed a striking distinction between the eukaryotic and bacterial PP2C-like phosphatases in motif II, where a histidine residue (His62 in human PP2Ca) is conserved in more than 90% of sequences, but not observed in the bacterial group. The bacterial PP2C group contains a methionine (at the corresponding position) in the majority of the cases (70%). This histidine residue is involved in the formation of a beta hairpin in the crystal structure of human PP2C [91]. Furthermore, His62 is proposed to act as a general acid for PP2C catalysis [92]. Both amino acids lie in the proximity of the phosphate-binding domain, but at this time it is unclear how the difference at this position would contribute to the overall structure and function of the two PP2C groups. Nonetheless, the large number of diverse PP2C-like phosphatases in this dataset allowed us to identify a previously unrecognized key difference between bacterial and eukaryotic PP2Cs.

Bacterial genes that perform closely related functions can be organized in close proximity to each other and often in functional units. Linked Ser/Thr kinase-phosphatase genetic units have been described in several bacterial species, including *Streptococcus pneumoniae, Bacillus subtilis,* and *Mycobacterium tuberculosis* [93–96]. Two major neighboring clusters are found to be associated with the set of PP2C-like phosphatases in the GOS bacterial group. We observed that one of these clusters contained a protein serine/threonine kinase domain as its most common Pfam domain. An additional neighboring cluster found to be associated with the GOS set of bacterial PP2Cs was identified as a set of sequences containing a PASTA (penicillin-binding protein and serine/threonine kinase–associated) domain. This domain is unique to bacterial species, and is believed to play important roles in regulating cell wall biosynthesis [97].

Our identification of a conserved group of unique PP2C-like phosphatases in the GOS dataset significantly increases the number and diversity of this enzyme family. This analysis of the NCBI-nr, PG ORFs, TGI-EST ORFs, and ENS datasets along with the sequences obtained from the GOS dataset significantly increases the overall number of PP2C-like sequences from that estimated just a year ago [98]. The presence of genes encoding bacterial serine/threonine kinase domains located adjacent to PP2Cs in the GOS data supports the notion that the process of reversible phosphorylation on Ser/Thr residues controls important physiological processes in bacteria.

### Proteases in GOS Data

Proteases are a group of enzymes that degrades other proteins and, as such, plays important roles in all organisms [99]. On the basis of their catalysis mechanism, proteases are divided into six distinct catalytic types: aspartic, cysteine, metallo, serine, threonine, and glutamic proteases [99]. They differ from each other by the presence of specific amino acids in the active site and by their mode of action. The MEROPS database [100] is a comprehensive source of information for this large divergent group of sequences and provides a widely accepted classification of proteases into families, based on the amino acid sequence comparison, and then into clans based on the similarity of their 3-D structures.

We identified 222,738 potential proteases in the GOS dataset based on similarity to sequences in MEROPS (see Materials and Methods). According to our clustering method, 95% of these sequences are grouped into 190 clusters, with each cluster on the average containing more than 1,100 GOS sequences. These sequences were compared to proteases in NCBI-nr. There are groups of proteases in NCBI-nr that are highly redundant. For example, there are a large number of viral proteases from HIV-1 and hepatitis C viruses that dominate the NCBI-nr protease set. Thus, we computed a nonredundant set of NCBI-nr proteases and, for the sake of consistency, a nonredundant set of proteases from the GOS set using the same parameters. The majority of proteases in both sets are dominated by cysteine, metallo, and serine proteases. The GOS dataset is dominated by proteases belonging to the bacterial kingdom. That is not surprising, given the filter sizes used to collect the samples. In NCBI-nr the proteases are more evenly distributed between the bacterial and the eukaryotic kingdoms.

Our comparison of the protease clan distribution of the bacterial sequences in the NCBI-nr and GOS sets reveals that the distribution of clans is very similar for metallo- and serine proteases. However, the distribution of clans in aspartic and cysteine proteases is different in the two datasets. Among aspartic proteases, the most visible difference is the increased ratio of proteases of the AC clan and the decreased ratio in the AD clan. Proteases in the former clan are involved in bacterial cell wall production, while those in the latter clan are involved in pilin maturation and toxin secretion [99]. Among cysteine proteases, the most apparent is the decrease in the CA clan and an increase in the number of proteases from the PB(C) clan. Bacterial members of the CA clan are mostly involved in degradation of bacterial cell wall components and in various aspects of biofilm formation [99]. It is possible that both activities are less important for marine bacteria present in surface water. Proteases from the PB(C) clan are involved in activation (including self-activation) of enzymes from acetyltransferase family. In fungi this family is involved in penicillin synthesis, while their function in bacteria is unknown [99].

We were unable to detect any caspases (members of the CD clan) in the GOS data. This is consistent with the apoptotic cell death mechanism being present only in multicellular eukaryotes, which, based on the filter sizes, are expected to be very rare in the GOS dataset.

### Metabolic Enzymes in the GOS Data

To gain insights into the diversity of metabolism of the organisms in the sea, we studied the abundance and diversity of glutamine synthetase (GS) and ribulose 1,5-bisphosphate carboxylase/oxygenase (RuBisCO), two key enzymes in nitrogen and carbon metabolism.

GS is the central player of nitrogen metabolism in all organisms on earth. It is one of the oldest enzymes in evolution [101]. It converts ammonia and glutamate into glutamine that can be utilized by cells. GS can be classified into three types based on sequence [101]. Type I has been found only in bacteria, and it forms a dodecameric structure [102,103]. Type II has been found mainly in eukaryotes, and in some bacteria. Type III GS is less well studied, but has been found in some anaerobic bacteria and cyanobacteria. There are 18 active site residues in both bacterial and eukaryotic GS that play important roles in binding substrates and catalyzing the enzymatic reactions [104].

We found 9,120 GS and GS-like sequences in the GOS data (Table 10). Using profile HMMs [41,105] constructed from known GS sequences of different types, we were able to classify 4,350 sequences as type I GS, 1,021 sequences as type II GS, and 469 sequences as type III GS (see Materials and Methods).

The number of type II GS sequences found in the GOS data is surprisingly high, since previously type II GS were considered to be mainly eukaryotic and very few eukaryotic organisms were expected to be included in the GOS sequencing (Figure 7C and Materials and Methods). We used gene neighbor analysis to classify the origin of GS genes by the nature of other proteins found on the same scaffold. Using this approach, most of the neighboring genes of the type II GS in the GOS data are identified as bacterial genes. The neighboring genes of the type II GS include nitrogen regulatory protein PII, signal transduction histidine kinase, NH_3_-dependent NAD^+^ synthetase, A/G-specific adenine glycosylase, coenzyme PQQ synthesis protein c, pyridoxine biosynthesis enzyme, aerobic-type carbon monoxide dehydrogenase, etc. We were able to assign more than 90% of the type II GS sequences in the GOS data to bacterial scaffolds based on a BLAST-based kingdom assignment method (see Materials and Methods). Both neighboring genes and kingdom assignments suggest that most of the type II GS sequences in the GOS data come from bacterial organisms. In comparison, the same type II GS profile HMM detects only 12 putative type II GS sequences from the PG dataset of 222 prokaryotic genomes. Within these, there are only seven unique type II GS sequences and six unique bacterial species represented. The reason why bacteria in the ocean have so many type II GS genes is unclear.

Two hypotheses have been raised to explain the origin of type II GS in bacterial genomes: lateral gene transfer from eukaryotic organisms [106] and gene duplication prior to the divergence of prokaryotes and eukaryotes [101]. The type II GS sequences in the predominantly bacterial GOS data are not only abundant, but also diverse and divergent from most of known eukaryotic GS sequences (Figure 7C). This makes the hypothesis of lateral gene transfer less favorable. If the GS gene duplication preceded the prokaryote–eukaryote divergence according to the gene duplication hypothesis, it is possible that many oceanic organisms retained type II GS genes during evolution.

Interestingly, we found 19 cases where a type I GS gene is adjacent to a type II GS gene on the same scaffold. Both GS genes seem to be functional based on the high degree of conservation of active site residues. The same gene arrangement was observed previously in Frankia alni CpI1 [107]. The functional significance of maintaining two types of GS genes adjacent to one another in the genome remains to be elucidated. Most of the sequences of these GS genes are highly similar. We examined the geographic distribution of these adjacent GS sequences across all the GOS samples. They are mainly found in the samples taken from two sites. Their geographic distribution is significantly different from the distributions of types I and II GS across the samples. The high sequence similarity among the adjacent GS pairs and their geographic distribution suggest that these adjacent GS sequences may come from only a few closely related organisms. This is consistent with the protein sequence tree of type II GS, where the type II GS sequences from the GS gene pairs mainly reside in two distinct branches (Figure 7C).

The active site residues are very well conserved in all GS sequences in the GOS data, except one residue, Y179, which coordinates the ammonium-binding pocket. We observed substitutions of Y179 to phenylalanine in about half of the type II GS sequences. The activity of type I GS in some bacteria is regulated by adenylylation at residue Tyr397. In the GOS data, Tyr397 is relatively conserved in type I GS, with variations to phenylalanine and tryptophan in about half of the sequences. This indicates that the activity of some of the type I GS is not regulated by adenylylation, as shown previously in some Gram-positive bacteria [108,109].

RuBisCO is the key enzyme in carbon fixation. It is the most abundant enzyme on earth [110] and plays an important role in carbon metabolism and CO_2_ cycle. RuBisCO can be classified into four forms. Form I has been found in both plants and bacteria, and has an octameric structure. Form II has been found in many bacteria, and it forms a dimer in *Rhodospirillum rubrum.* Form III is mainly found in archaea, and forms various oligomers. Form IV, also called the RuBisCO-like protein (RLP), has been recently discovered from bacterial genome-sequencing projects [111,112]. RLP represents a group of proteins that do not have RuBisCO activity, but resemble RuBisCO in both sequence and structure [111,113]. The functions of RLPs are largely unknown and seem to differ from each other.

Contrary to the large number of GS sequences, we identified only 428 sequences homologous to the RuBisCO large subunit in the GOS data. The small number of RuBisCO sequences may partly be due to the fact that larger-sized bacterial organisms were not included in the sequencing because of size filtering. However, it could also indicate that CO_2_ is not the major carbon source for these sequenced ocean organisms.

The RuBisCO homologs in the GOS data are more diverse than the currently known RuBisCOs (Figure 7D, Materials and Methods). Six of 19 active site residues—N123, K177, D198, F199, H327, and G404—are not well conserved in all sequences, suggesting that the proteins with these mutations may have evolved to have new functions, such as in the case of RLPs. From the studies of the RLPs from Chlorobium tepidum and B. subtilis [111,114], it has been shown that the active site of RuBisCO can accommodate different substrates and is potentially capable of evolving new catalytic functions [113,114]. On the other hand, two sequence motifs, helices αB and α8, that are not involved in substrate binding and catalytic activity are well conserved in the GOS RuBisCO sequences. The higher degree of conservation of these nonactive site residues than that of active site residues suggests that these motifs are important for their structure, function, or interaction with other proteins.

We found 47 (31 at 90% identity filtering) GOS sequences in the branch with known RLP sequences in a phylogenetic tree of RuBisCO (Figure 7D). In this phylogenetic tree, in addition to the clades for each of the four forms of RuBisCO, there are also new groups of 65 (58 at 90% identity filtering) GOS sequences that do not cluster with any known RuBisCO sequences. This indicates that there could be more than one type of RuBisCO-like protein existing in organisms. The novel groups of RuBisCO homologs in the GOS data also suggest that we have not fully explored the entire RuBisCO family of proteins (Figure 7D).

### GOS Data and Remote Homology Detection

**Figure 8:**
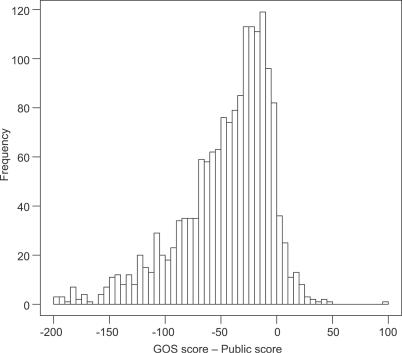
Distribution of Average HMM Score Difference between GOS and Public (NCBI-nr, MG, TGI-EST, and ENS) Only matches to the full length of an HMM are considered, and only HMMs that have at least 100 matches to each of GOS and public databases are considered. This results in 1,686 HMMs whose average scores to GOS and public databases are considered. The mean of the distribution is −50, showing that GOS sequences tend to score lower than sequences in public, thereby reflecting diversity compared to sequences in public.

The addition of GOS sequences may help greatly in defining the range and diversity of many known protein families, both by addition of many new sequences and by the increased diversity of GOS sequences. Our comparison of HMM scores for GOS sequences with those from the other four datasets shows that GOS sequences consistently tend to have lower scores, which indicates additional diversity from that captured in the original HMM (Figure 8). The addition of GOS data into domain profiles may broaden the profile and allow it to detect additional remote family members in both GOS and other datasets. As a trial, we rebuilt the Pfam model PF01396, which describes a zinc finger domain within bacterial DNA topoisomerase. The original model finds 821 matches to 481 proteins in NCBI-nr. Our model that includes GOS sequences reveals 1,497 matches to 722 sequences, an increase of 50% in sequences and 82% in domains (most topoisomerases have three such domains, of which one is divergent and difficult to detect). Of these new matches, 104 are validated by the presence of additional topoisomerase domains, or they are annotated as topoisomerase, while most others are unannotated or similar to other DNA-modifying enzymes not previously thought to have zinc finger domains.

HMM profiles can be further exploited by using matches beyond the conservative trusted cutoff (TC) used in this study. For instance, the Pfam for the poxvirus A22 protein family has no GOS matches above the TC, but 137 matches with *E-*values of 1 × 10^−3^ to 1 × 10^−10^, containing a short conserved motif overlap with A22 proteins. Alignment of these matches shows an additional two short motifs in common with A22, establishing their homology, and using a profile HMM, we found a total of 269 family members in GOS and eight family members in NCBI-nr. Many members of this new family are surrounded by other novel clusters, or are in putative viral scaffolds, suggesting that these weak matches are an entry point into a new clade of viruses.

### ORFans with Matches in GOS Data

Further evidence of the diversity added by GOS sequences is provided by their matches to ORFans. ORFans are sequences in current protein databases that do not have any recognizable homologs [117]. ORFan sequences (discounting those that may be spurious gene predictions) represent genes with organism-specific functions or very remote homologs of known families. They have the potential to shed light on how new proteins emerge and how old ones diversify.

**Figure 9:**
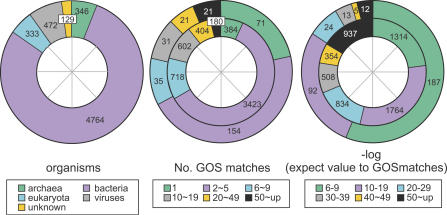
Pie Chart of ORFans That Had GOS Matches ORFans are grouped by organism (left), number of their GOS matches (middle), and the lowest *E-*value to their GOS matches in negative logarithm form (right). For both middle and right charts, inner and outer circles represent noneukaryotic and eukaryotic ORFans, respectively. From the middle chart it is seen that 626 (= 404 + 180 + 21 + 21) ORFans form significant protein families with ≥20 GOS matches.

We identified 84,911 ORFans (5,538 archaea, 35,292 bacteria, 37,427 eukaryotic, 5,314 virus, and 1,340 unclassified) from the NCBI-nr dataset using CD-HIT [116,117] and BLAST (see Materials and Methods). Of these, 6,044 have matches to GOS sequences using BLAST (*E-*value ≤1 × 10^−6^). Figure 9 shows the distribution of the matched ORFans grouped by organisms, number of their GOS matches, and the lowest *E-*value of the matches. We found matches to GOS sequences for 13%, 6.3%, 0.89%, and 8.9% of bacterial, archaeal, eukaryotic, and viral ORFans, respectively. While most of these ORFans have very few GOS matches, 626 of them have ≥20 GOS matches. The similarities between GOS sequences and eukaryotic ORFans are much weaker than those between GOS sequences and noneukaryotic ORFans. The average sequence identity between eukaryotic ORFans and their closest GOS matches is 38%. This is 6% lower than the identity between noneukaryotic ORFans and their closest GOS matches.

The ORFans that match GOS sequences are from approximately 600 organisms. Table 11 lists the 20 most populated organisms. Out of the 6,044 matched ORFans, approximately 2,000 are from these 20 organisms. For example, Rhodopirellula baltica SH 1, a marine bacterium, has 7,325 proteins deposited in NCBI-nr. We identified 1,418 ORFans in this organism, of which 322 have GOS matches. Another interesting example in this list is *Escherichia coli.* Although there are >20 different strains sequenced, 168 ORFans are identified in strain CFT073, and 67 of them have GOS matches. The only eukaryotic organism in this list is Candida albicans SC5314, a fungal human pathogen, which has 49 ORFans with GOS matches.

**Table 11:**
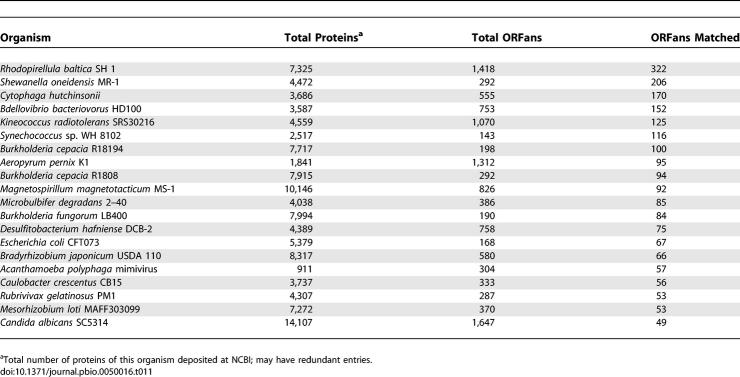
Top 20 Organisms with Most ORFans Matched by GOS

We examined a small but interesting subset of the ORFans that have 3-D structures deposited in PDB. Out of 65 PDB ORFans, GOS matches for eight of them are found (see Supporting Information for their PDB identifiers and names). They include four restriction endonucleases, three hypothetical proteins, and a glucosyltransferase.

**Figure 10:**
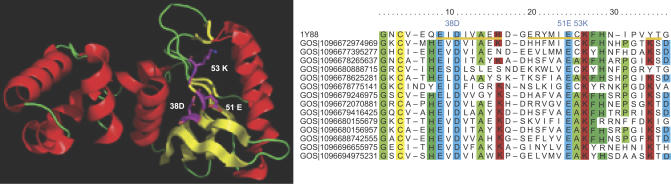
Structure and GOS Homologs of Hypothetical Protein AF1548 Yellow bars represent β-strands. Highlighted are predicted catalytic residues: 38D, 51E, and 53K.

GOS sequences can play an important role in identifying the functions of existing ORFans or in confirming protein predictions. For example, we found that the hypothetical protein AF1548, which is a PDB ORFan, has matches to 16 GOS sequences. A PSI-BLAST search with AF1548 as the query against a combined set of GOS and NCBI-nr identified several significant restriction endonucleases after three iterations. With the support of 3-D structure and multiple sequence alignment of AF1548 and its GOS matches, we predict that AF1548 along with its GOS homologs are restriction endonucleases (Figure 10). When combined with an established consensus of active sites of the related endonucleases families [118], we predicted three catalytic residues.

### Genome Sequencing Projects and Protein Exploration

**Figure 11:**
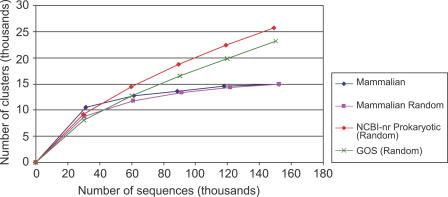
Rate of Cluster Discovery for Mammals Compared to That for Microbes The *x*-axis denotes the number of sequences (in thousands), and the *y*-axis denotes the number of clusters (in thousands). Five mammalian genomes are considered for the “Mammalian” dataset, and the plot shows the number of clusters that are hit when each additional genome is added. For the “Mammalian Random” dataset, the order of the sequences from the “Mammalian” dataset is randomized. For the NCBI-nr prokaryotic and GOS datasets, random subsets of size similar to that of the mammalian set are considered.

With respect to protein exploration and novel family discovery, microbial sequencing offers more promise compared to sequencing more mammalian genomes. This is illustrated by Figure 11 **,** where the number of clusters that protein predictions from various finished mammalian genomes fall into was compared to the number of clusters that similar-sized random subsets of microbial sequences fall into (see Materials and Methods). As the figure shows, the rate of protein family discovery is higher for microbes than for mammals. Indeed, the rate of new family discovery is plateauing for mammalian sequences. This is not surprising, as mammalian divergence from a common ancestor is much more recent than microbial divergence from a common ancestor, which suggests that mammals will share a larger core set of less-diverged proteins. Microbial sequencing is also more cost effective than mammalian sequencing for acquiring protein sequences because microbial protein density is typically 80%–90% versus 1%–2% for mammals. This could be addressed with mammalian mRNA sequencing, but issues with acquiring rarely expressed mRNAs would need to be considered. There are, of course, other reasons to sequence mammalian genomes, such as understanding mammalian evolution and mammalian gene regulation.

### Conclusions

The rate of protein family discovery is approximately linear in the (current) number of protein sequences. Additional sequencing, especially of microbial environments, is expected to reveal many more protein families and subfamilies. The potential for discovering new protein families is also supported by the GOS diversity seen at the nucleotide level across the different sampling sites [30]. Averaged over the sites, 14% of the GOS sequence reads from a site are unique (at 70% nucleotide identity) to that site [30].

The GOS data provides almost complete coverage of known prokaryotic protein families. In addition, it adds a great deal of diversity to many known families and offers new insights into the evolution of these families. This is illustrated using several protein families, including UV damage–repair enzymes, phosphatases, proteases, glutamine synthetase, RuBisCO, RecA (unpublished data), and kinases [77]. Only a handful of protein families have been examined thus far, and many thousands more remain to be explored.

The protein analysis presented indicates that we are far from exploring the diversity of viruses. This is reflected in several of the analyses. The GOS-only clusters show an overrepresentation of sequences of viral origin. In addition, our domain analysis using HMM profiling shows a lower Pfam coverage of the GOS sequences in the viral kingdom compared to the other kingdoms. At least two of the protein families we explored in detail (UV repair enzymes and glutamine synthetase) contain abundant new viral additions. The extraordinary diversity of viruses in a variety of environmental settings is only now beginning to be understood [57,119–121]. A separate analysis of GOS microbial and viral sequences (unpublished data) shows that multiple viral protein clusters contain significant numbers of host-derived proteins, suggesting that viral acquisition of host genes is quite widespread in the oceans.

Data generated by this GOS study and similar environmental shotgun sequencing studies present their own analysis challenges. Methods for various analyses (e.g., sequence alignment, profile construction, phylogeny inference, etc.) are generally designed and optimized to work with full sequences. They have to be tailored to analyze the mostly fragmentary sequences that are generated by these projects. Nevertheless, these data are a valuable source of new discoveries. These data have the potential to refine old hypotheses and make new observations about proteins and their evolution. Our preliminary exploration of the GOS data identified novel protein families and also showed that many ORFan sequences from current databases have homologs in these data. The diversity added by GOS data to protein families also allows for the building of better profile models and thereby improves remote homology detection. The discovery of kingdom-crossing protein families that were previously thought to be kingdom-specific presents evidence that the GOS project has excavated proteins of more ancient lineage than that previously known, or that have undergone lateral gene transfer. This is another example of how metagenomics studies are changing our understanding of protein sequences, their evolution, and their distribution across the various forms of life and environments. Biases in the currently published databases due to oversampling of some proteins or organisms are illuminated by environmental surveys that lack such biases. Such knowledge can help us make better predictions of the real distribution patterns of proteins in the natural world and indicate where increased sampling would be likely to uncover new families or family members of tremendous diversity (such as in the viral kingdom).

These data have other significant implications for the fields of protein evolution and protein structure prediction. Having several hundreds or even tens of thousands of diverse proteins from a family or examples of a specific protein fold should provide new approaches for developing protein structure prediction models. Development of algorithms that consider the alignments of all these family members/protein folds and analyze how amino acid sequence can vary without significantly altering the tertiary structure or function may provide insights that can be used to develop new ab inito methods for predicting protein structures. These same datasets could also be used to begin to understand how a protein evolves a new function. Finally, this large database of amino acid sequence data could help to better understand and predict the molecular interactions between proteins. For example, they may be used to predict the protein–protein interactions so critical for the formation of specific functional complexes within cells.

The GOS data also have implications for nearly all computational methods relying on sequence data. The increase in the number of known protein sequences presents challenges to many algorithms due to the increased volume of sequences. In most cases this increase in sequence data can be compensated for with additional CPU cycles, but it is also a foreshadowing of times to come as the pace of large-scale sequence-collecting accelerates. A related challenge is the increase in the diversity of protein families, with many new divergent clades present. With more protein similarity relationships falling into the twilight zone overlapping with random sequence similarity, the number of false positives for homology detection methods increases, making the true relationships more difficult to identify. Nevertheless, a deeper knowledge of protein sequence and family diversity introduces unprecedented opportunities to mine similarity relationships for clues on molecular function and molecular interactions as well as providing much expanded data for all methods utilizing homologous sequence information data.

The GOS dataset has demonstrated the usefulness of large-scale environmental shotgun sequencing projects in exploring proteins. These projects offer an unbiased view of proteins and protein families in an environmental sample. However, it should be noted that the GOS data reported here are limited to mostly ocean surface microbes. Even with this targeted sampling a tremendous amount of diversity is added to known families, and there is evidence for a large number of novel families. Additional data from larger filter sizes (that will sample more eukaryotes) coupled with metagenomic studies of different environments like soil, air, deep sea, etc. will help to achieve the ultimate goal of a whole-earth catalog for proteins.

## Materials and Methods

### Data description.

NCBI-nr [31,32] is the single largest publicly available protein resource and includes protein sequences submitted to SWISS-PROT (curated protein database) [122], PDB (a database of amino acid sequences with solved structures) [123], PIR (Protein Information Resource) [124], and PRF (Protein Research Foundation). In addition, NCBI-nr also contains protein predictions from DNA sequences from both finished and unfinished genomes in GenBank [125], EMBL [126], and DNA Databank of Japan (DDBJ) [127]. The nonredundancy in NCBI-nr is only to the level of distinct sequences, and any two sequences of the same length and content are merged into a single entry. NCBI-nr contains partial protein sequences and is not a fully curated database. Therefore it also contains contaminants in the form of sequences that are falsely predicted to be proteins.

Expressed sequence tag (EST) databases also provide the potential to add a great deal of information to protein exploration and contain information that is not well represented in NCBI-nr. To this end, assemblies of EST sequences from the TIGR Gene Indices [34], an EST database, were included in this study. To minimize redundancy, only EST assemblies from those organisms for which the full genome is not yet known, were included. The protein predictions on metazoan genomes that are fully sequenced and annotated were obtained by including the Ensembl database [35,36] in this study.

Both finished and unfinished sequences from prokaryotic genome projects submitted to NCBI were included. The protein predictions from the individual sequencing projects are submitted to NCBI-nr. Nevertheless, these genomes were included in this dataset both for the purpose of evaluating our approach and also for the purpose of identifying any proteins that were missed by the annotation process used in these projects.

Thus, for this study the following publicly available datasets, all downloaded on February 10, 2005—NCBI-nr, PG, TGI-EST, and ENS—were used. The organisms in the PG set and the TGI-EST set are listed in Protocol S1.

### Assembly of the GOS dataset.

Initial assembly (construction of “unitigs”) was performed so that only overlaps of at least 98% DNA sequence identity and no conflicts with other overlaps were accepted. False assemblies at this phase of the assembler are extremely rare, even in the presence of complex datasets [37,128]. Paired-end (also known as mate-pair) data were then used to order, orient, and merge unitigs into the final assemblies, but only when two mate pairs or a single mate pair and an overlap between unitigs implied the same layout. In one respect, mate pair data was used more aggressively than is typical in assembly of a single genome in that depth-of-coverage information was largely ignored [10]. This potentially allows chimeric assemblies through a repeat within a genome or through an ortholog between genomes. Thus, a conclusion that relies on the correctness of a single assembly involving multiple unitigs should be considered tentative until the assembly can be confirmed in some way. Assemblies involved in key results in this paper were subjected to expert manual review based on thickness of overlaps, presence of well-placed mate pairs across thin overlaps or across gaps between contigs, and consistency of depth of coverage.

### Data release and availability.

All the GOS protein predictions will be submitted to GenBank. In addition, all the data supporting this paper, including the clustering and the various analyses, will be made publicly available via the CAMERA project (Community Cyberinfrastructure for Advanced Marine Microbial Ecology Research and Analysis; http://camera.calit2.net), which is funded by the Gordon and Betty Moore Foundation.

### All-against-all BLASTP search.

We used two sets of computer resources. At the J. Craig Venter Institute, 125 dual 3.06-GHz Xeon processor systems with 2 Gb of memory per system were used. Each system had 80 GB local storage and was connected by GBit ethernet with storage area network (SAN) I/O of ~24 GBit/sec and network attached storage (NAS) I/O of ~16 GBit/sec. A total of 466,366 CPU hours was used on this system. In addition, access to the National Energy Research Scientific Computing Center (NERSC) Seaborg computer cluster was available, including 380 nodes each with sixteen 375-MHz Power3 processors. The systems had between 16 GB and 64 GB of memory. Only 128 nodes were used at a time. A total of 588,298 CPU hours was used on this system. The dataset of 28.6 million sequences was searched against itself in a half-matrix using NCBI BLAST [38] with the following parameters: -F “m L” -U T -p blastp -e 1 × 10^−10^ -z 3 × 10^9^ -b 8000 -v 10. In this paper, *similarity* of an alignment is defined to be the fraction of aligned residues with a positive score according to the BLOSUM62 substitution matrix [129] used in the BLAST searches.

### Identification of nonredundant sequences.

Given a set of sequences *S* and a threshold *T*, a nonredundant subset *S′* of *S* was identified by first partitioning *S* (using the threshold *T*) and then picking a representative from each partition. The set of representatives constitutes the nonredundant set *S′*. The process was implemented using the following graph-theoretic approach. A directed graph *G* = (*V, E*) is constructed with vertex set *V* and edge set *E*. Each vertex in *V* represents a sequence from *S*. A directed edge (*u*,*v*) ∈ *E* if sequence *u* is longer than sequence *v* and their sequence comparison satisfies the threshold *T;* for sequences of identical length, the sequence with the lexicographically larger id is considered the longer of the two. Note that *G* does not have any cycles. Source vertices (i.e., vertices with no in-degree) are sorted in decreasing order of their out-degrees and (from largest out-degree to smallest) processed in this order. A source vertex *u* is processed as follows: mark all vertices that have not been seen before and are reachable from vertex *u* as being redundant and mark vertex *u* as their representative.

We used two thresholds in this paper, 98% similarity and 100% identity. The former was used in the first stage of the clustering and the later was used in the HMM profile analysis. For the 98% similarity threshold, two sequences satisfy the threshold if the following three criteria are met: (1) similarity of the match is at least 98%; (2) at least 95% of the shorter sequence is covered by the match; and (3) (match score)/(self score of shorter sequence) ≥ 95%.

For the 100% identity threshold, two sequences satisfy the threshold if their match identity is 100%.

### Description of the clustering algorithm.

The starting point for the clustering was the set of pairwise sequence similarities identified using the all-against-all BLASTP compute. Because of both the volume and nature of the data, the clustering was carried out in four steps: redundancy removal, core set identification, core set merging, and final recruitment.

A set of nonredundant sequences (at 98% similarity) was identified using the procedure given in Materials and Methods (Identification of nonredundant sequences). Only the nonredundant sequences were considered in further steps of the clustering process.

The aim of the core set identification step was to identify *core sets* of highly related sequences. In graph-theoretic terms, this involves looking for dense subgraphs in a graph where the vertices correspond to sequences and an edge exists between two sequences if their sequence match satisfies some reasonable threshold (for instance, 40% similarity match over 80% of at least one sequence and are clearly homologous based on the BLAST threshold). Dense subgraphs were identified by using a heuristic. This approach utilizes *long* edges. These are edges where the match threshold is computed relative to the longer sequence. This was done to prevent, as much as possible, unrelated proteins from being put into the same core set. If all the sequences were full length, using long edges would have offered a good solution to keeping unrelated proteins apart. However, the situation here is complicated by the presence of a large amount of fragmentary sequence data of varying lengths. This was dealt with somewhat by working with rather stringent match thresholds and a two-stage process to identify the core sets. We used the concept of *strict* long edges and *weak* long edges. A strict long edge exists between two vertices (sequences) if their match has the following properties: (1) 90% of the longer sequence is involved in the match; (2) the match has 70% similarity; and (3) the score of the match is at least 60% of the self-score of the longer sequence. A weak long edge exists between two vertices (sequences) if their match has the following properties: (1) 80% of the longer sequence is involved in the match; (2) the match has 40% similarity; and (3) the score of the match is at least 30% of the self-score of the longer sequence. Core set identification had two substages: *large core initialization* and *core extension.* The large core initialization step identified sets of sequences where these sets were of a reasonable size and the sequences in them were very similar to each other. Furthermore, these sets could be extended in the core extension step by adding related sequences. In the large core initialization step, a directed graph *G* was constructed on the sequences using strict long edges, with each long edge being directed from the longer to the shorter sequence. For each vertex *v* in *G*, let *S*(*v*) denote the friends set of *v* consisting of *v* and all neighbors that *v* has an out-going edge to.

Initially all the vertices in G are unmarked. Consider the set of all friends sets in the decreasing order of their size. For *S*(*v*) that is currently being considered, do the following: (1) initialize *seed set A* = *S*(*v*); (2) while there exists some *v*′ such that |*S*(*v*) ∩ *S*(*v*′)| ≥ *k*, set *A* = *A* ∪ *S*(*v*′). (Note: *k* = 10 is chosen); (3) output set *A* and mark all vertices in *A*; and (4) update all friends sets to contain only unmarked vertices.

In the core extension step, we constructed a graph *G* using weak long edges. All vertices in seed sets (computed from the large core initialization step) were marked and the rest of the vertices unmarked. Each seed set was then greedily extended to be a core set by adding a currently unmarked vertex that has at least *k* neighbors (*k* = 10 is chosen) in the set; the added vertex was marked. After this process, a clique-finding heuristic was used to identify smaller cliques (of size at most *k* − 1) consisting of currently unmarked vertices; these were also extended to become core sets. A final step involved merging the computed core sets on the basis of weak edges connecting them.

In the core set merging step, we constructed an FFAS (Fold and Function Assignment System) profile [39] for each core set using the longest sequence in the core set as query. FFAS was then used to carry out profile–profile comparisons in order to merge the core sets into larger sets of related sequences. Due to computational constraints imposed by the number of core sets, profiles were built on only core sets containing at least 20 sequences.

Final recruitment involved constructing a PSI-BLAST profile [40] on core sets of size 20 or more (using the longest sequence in the core set as query) and then using PSI-BLAST (–z 1 × 10^9^, –e 10) to recruit as yet unclustered sequences or small-sized clusters (size less than 20) to the larger core sets. For a sequence to be recruited, the sequence–profile match had to cover at least 60% of the length of the sequence with an *E-*value ≤ 1 × 10^−7^. In a final step, unclustered sequences were recruited to the clusters using their BLAST search results. A length-based threshold was used to determine if the sequence is to be recruited.

### Identification of clusters containing shadow ORFs.

A well-known problem in predicting coding intervals for DNA sequences is shadow ORFs. The key requirement that coding intervals not contain in-frame stop codons requires that coding intervals be subintervals of ORFs. Long ORFs are therefore obvious candidates to be coding intervals. Unfortunately, the constraints on the coding interval to be an ORF often cause subintervals and overlapping intervals of the coding interval to also be ORFS in one of the five other reading frames (two on the same strand and three on the opposite strand). These coincidental ORFs are called shadow ORFs since they are found in the shadow of the coding ORF. In rare cases (and more frequently in certain viruses) coding intervals in different reading frames can overlap but usually only slightly. Overwhelmingly distinct coding intervals do not overlap. However, this constraint is not as strict for ORFs that contain a coding interval, as the exact extent of the coding interval is not known. Prokaryotes predominate in these data and are the focus of the ORF predictions. Their 3′ end of an ORF is very likely to be part of the coding interval because a stop codon is a clear signal for the termination of both the ORF and the coding interval (this signal could be obscured by frameshift errors in sequencing). The 5′ end is more problematic because the true start codon is not so easily identified and so the longest ORF with a reasonable start codon is chosen and this may extend the ORF beyond the true coding interval. For this reason different criteria were set for when ORFs have a significant overlap depending on the orientation (or the 5′ or 3′ ends) of the ORFs involved. Two ORFs on the same strand are considered overlapping if their intervals overlap by at least 100 bp. Two ORFs that are on the opposite strands are considered overlapping either if their intervals overlap by at least 50 bp and their 3′ ends are within each others intervals, or if their intervals overlap by at least 150 bp and the 5′ end of one is in the interval of the other.

ORFs for coding intervals are clustered based on sequence similarity. In most cases this sequence similarity is due to the ORFs evolving from a common ancestral sequence. Due to functional constraints on the protein being coded for by the ORF, some sequence similarity is retained. There are no known explicit constraints on the shadow ORFs to constrain drift from the ancestral sequences. However, the shadow ORFs still tend to cluster together for some obvious reasons. The drift has not yet obliterated the similarity. There are implicit constraints due to the functional constraints on the overlapping coding ORF. There are also other possible unknown functional constraints beyond the coding ORF. At first it was surmised that within shadow ORF clusters the diversity should be higher than for the coding ORF, but this did not prove to be a reliable signal. The apparent problem is that the shadow ORFs tend to be fractured into more clusters due to the introduction of stop codons that are not constrained because the shadow ORFs are noncoding. What rapidly became apparent is that the most reliable signal that a cluster was made up of shadow ORFs is that the cluster was smaller than the coding cluster containing the ORFs overlapping the shadow ORFs.

The basic rule for labeling a cluster as a shadow ORF cluster is that the size of the shadow ORF cluster is less than the size of another cluster that contained a significant proportion of the overlapping ORFs for the shadow ORF cluster. A specific set of rules was used to label shadow ORF clusters based on comparison to other clusters that contained ORFs overlapping ORFS in the shadow ORF cluster (called the overlapping cluster for this discussion). First, the overlapping cluster cannot be the same cluster as the shadow ORF cluster (there are sometimes overlapping ORFs within the same cluster due to frameshifts). Second, both the redundant and nonredundant sizes of the shadow ORF cluster must be smaller than the corresponding sizes of the overlapping cluster. Third, at least one-third of the shadow ORFs must have overlapping ORFs in the overlapping cluster. Fourth, less than one-half of the shadow ORFs are allowed to contain their overlapping ORFs (this test is rarely needed but did eliminate the vast majority of the very few obvious false positives that were found using these rules). Finally, the majority of the shadow ORFs that overlapped must overlap by more than half their length.

When using this rule, 1,274,919 clusters were labeled as shadow ORF clusters, and 6,570,824 singletons were labeled as shadow ORFs. The rules need to be somewhat conservative so as not to eliminate coding clusters. To test these rules, clusters containing at least two NCBI-nr sequences were examined. Two sequences were used instead of one because occasional spurious shadow ORFs have been submitted to NCBI-nr. There were 989 shadow ORF clusters containing at least two NCBI-nr sequences and with more than one-tenth as many NCBI-nr sequences as the overlapping cluster. This was 0.86% of all clusters (114,331 in total) with at least two NCBI-nr sequences. Of these 989, a few were obvious mistakes, and the others involved very few NCBI-nr sequences of dubious curation, such as “hypothetical.” Just to be conservative, all of these 989 clusters were rescued and not labeled as shadow ORF clusters.

### Ka/Ks test to determine if sequences in a cluster are under selective pressure.

For a cluster containing conserved but noncoding sequences, it is expected that there is no selection at the codon level. We checked this by computing the ratio of nonsynonymous to synonymous substitutions (Ka/Ks test) [130,131] on the DNA sequences from which the ORFs in the cluster were derived. For most proteins, Ka/Ks ≪ 1, and for proteins that are under strong positive selection, Ka/Ks ≫ 1. A Ka/Ks value close to 1 is an indication that sequences are under no selective pressure and hence are unlikely to encode proteins [134,135]. Weakly selected but legitimate coding sequences can have a Ka/Ks value close to 1. These were identified to some extent by using a model in which different partitions of the codons experience different levels of selective pressure. A cluster was rejected only if no partition was found to be under purifying selection at the amino acid level.

The Ka/Ks test [130,131] was run only on those clusters (remaining after the shadow ORF filtering step) that did not contain sequences with HMM matches or have NCBI-nr sequences in them. Only the nonredundant sequences in a cluster were considered. Sequences in each of the clusters were aligned with MUSCLE [134]. For each cluster, a strongly aligning subset of sequences was selected for the Ka/Ks analysis. The codeml program from PAML [135,136] was run using model M0 to calculate an overall (i.e., branch- and position-independent) Ka/Ks value for the cluster. Clusters with Ka/Ks ≤ 0.5, indicating purifying selection and therefore very likely coding, were considered as passing the Ka/Ks filter. In addition, the remaining clusters were examined by running codeml with model M3. This partitioned the positions of the alignment into three classes that may be evolving differently (typically, a few positions may be under positive selection while the remainder of the sequence is conserved). A likelihood ratio test was applied to select clusters for which M3 explained the data significantly better than M0 [136]. If a cluster was thus selected, and if one of the resulting partitions had a Ka/Ks ≤ 0.5 and comprised at least 10% of the sequence, then that cluster was also considered as passing the Ka/Ks filter. All other clusters were marked as containing spurious ORFs.

### Statistics for the various stages of the clustering process

The number of sequences that remain after redundancy removal (at 98% similarity) for each dataset is given in Table 12. Recall that the size of a cluster is the number of nonredundant sequences in it.

**Table 12:**
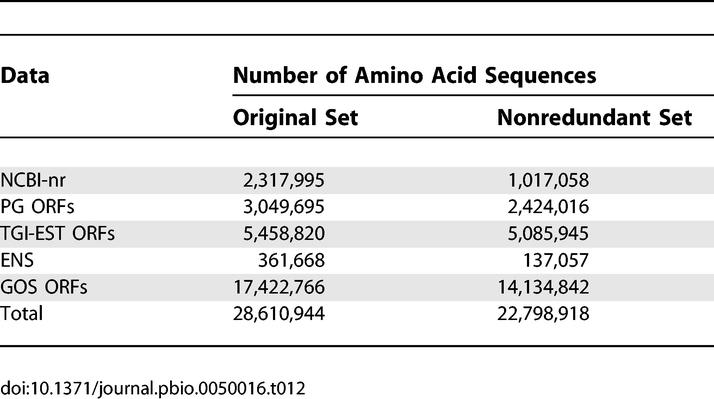
The Number of Sequences in NCBI-nr, PG ORFs, TGI-EST ORFs, ENS, and GOS ORFs prior to and after the Redundancy Removal Step of Our Clustering

Number of core sets of size two or more totals 1,586,454; number of nonredundant sequences in core sets of size two or more totals 8,337,256; and total number of sequences in core sets of size two or more is 12,797,641.

Total number of clusters after profile merging and (PSI-BLAST and BLAST) recruitment is 1,871,434; number of clusters of size two or more totals 1,388,287; number of nonredundant sequences in clusters of size two or more totals 11,494,078; total number of sequences in clusters of size two or more is 16,565,015.

The final clustering statistics (after shadow ORF detection and Ka/Ks tests) are as follows: number of clusters of size two or more totals 297,254; number of nonredundant sequences in clusters of size two or more totals 6,212,610; total number of sequences in clusters of size two or more is 9,978,637.

In the final BLAST recruitment step, a pattern was seen involving highly compositionally biased sequences that recruited unrelated sequences to clusters. This was reflected in the pre- and post-BLAST recruitment numbers, where the postrecruitment sizes were more than three to four times the size of the prerecruitment numbers. There were 75 such clusters, and these were removed.

### Searching sequences using profile HMMs.

The full set of 7,868 Pfam release 17 models was used, along with additional nonredundant profiles from TIGRFAM (1,720 of 2,443 profiles; version 4.1). HMM profiling was carried out using a TimeLogic DeCypher system (Active Motif, Inc., http://www.activemotif.com) and took 327 hours in total (on an eight-card machine). A sequence was considered as matching a Pfam (fragment model) if its sequence score was above the TC score for that Pfam and had an *E-*value ≤ 1 × 10^−3^. It was considered as matching a TIGRFAM if the match had an *E-*value ≤ 1 × 10^−7^.

### Evaluation of protein prediction via clustering.

Our evaluation of protein prediction via the clustering shows a very favorable comparison to currently used protein prediction methods for prokaryotic genomes. We used the PG dataset for this evaluation (Table 2). Of the 3,049,695 PG ORFs, 575,729 sequences (19%) were clustered (the *clustered set*). Of the 614,100 predictions made by the genome projects, 600,911 sequences could be mapped to the PG ORF set (the *submitted set*); 93% of the unmapped sequences were <60 aa (recall that the ORF calling procedure only produced ORFs of length ≥60 aa). The clustered set and submitted set had 493,756 ORFs in common. Of the 107,155 sequences that were only in the submitted set, 24,217 sequences (23%) had HMM matches. As with other unclustered HMM matches, most were weak or partial. These sequences had an average of only 48% of their lengths covered by HMMs. Of the remaining 82,938 sequences that did not have an HMM match, 13,724 (17%) were removed by the filters used, and the rest fell into clusters with only one nonredundant sequence (and thus were not labeled as predicted proteins by the clustering analysis). Based on NCBI-nr sequences in them, these clusters were mostly labeled as “hypothetical,” “unnamed,” or “unknown.” Our clustering method identified 81,973 ORFs not predicted by the genome projects, of which 16,042 (20%) were validated by HMM matches (with average HMM coverage of 69% of sequence length) and an additional 27,120 (33%) had significant BLAST matches (*E-*value ≤ 1 × 10^−10^) to sequences in NCBI-nr. Thus, if the submitted set is considered as truth, then protein prediction via clustering produces 493,756 true positives (TP), 81,973 false positives (FP), and 107,155 false negatives (FN), thereby having a sensitivity (TP/[TP + FN]) of 83% and specificity (TP/[TP + FP]) of 86%. However, if truth is considered as those sequences that are common to both the clustered and submitted sets in addition to those sequences with HMM matches, then our protein prediction method via clustering has 95% sensitivity and 89% specificity, while protein prediction by the prokaryotic genome projects has 97% sensitivity and 86% specificity.

### Evaluation of protein clustering.

We used Pfams to evaluate the clustering method in two ways. For both evaluations the clustering was restricted to only those sequences with Pfam matches. It should be kept in mind that there are redundancies among Pfams in that there can be more than one Pfam for a homologous domain family (for instance, the kinase domain Pfams—PF00069 protein kinase domain and PF07714 protein tyrosine kinase), and these redundancies can affect the evaluation statistics reported below.

For the first evaluation, each sequence was represented by the set of Pfams that match it. This is referred to as the *domain architecture* for a sequence. While Pfams provide a domain-centric view of proteins, the domain architecture attempts to approximate the full sequence-based approach used here, and thus could be used to shed light on the general performance of the clustering. We measured how often unrelated sequences were present in a given cluster. Two sequences were defined to be unrelated if their domain architectures each had at least one Pfam that was not present in the other's domain architecture. Note that this measure did not penalize the case when the domain architecture of one sequence was a proper subset of the domain architecture of the other sequence. This was done to allow fragmentary sequences in clusters to be included in the evaluation as well (and also because it is not always easy to determine whether an amino acid sequence is fragmentary or not). For each cluster, we computed the percentage of sequence pairs that are unrelated under this measure. A total of 92% of the clusters had at most 2% unrelated pairs. Then we carried out an assessment of how many instances of a given domain architecture appear in a single cluster. A total of 58% of the domain architectures were confined to single clusters (i.e., 100% of their occurence is in one cluster), and 88% of the domain architectures was such that >50% of their occurences is in one cluster.

**Figure 12:**
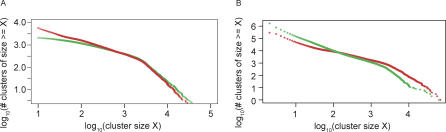
Log–Log Plots of Cluster Size Distributions The *x*-axis is logarithm of the cluster size *X* and the *y*-axis is the logarithm of the number of clusters of size at least *X;* logarithms are base 10. (A) Plot comparing the sizes of clusters produced by our clustering approach (red) to those of clusters produced by Pfams (green). The curves track each other quite well, with both of them having an inflection point around cluster size 2,500 (approximately 3.4 on the *x*-axis). Each sequence is assigned to the highest scoring Pfam that it matches. Two sequences that are assigned to the same Pfam can nevertheless be assigned to different clusters by the full-sequence–based clustering approach if they differ in the remaining portion. This is especially true for commonly occurring domains that are present in different multidomain proteins. Thus, there tends to be a larger number of big clusters in the Pfam approach as compared to the full-sequence–based approach. Hence, the green curve is above the red curve at the higher sizes. (B) Plot of the cluster size distributions for core sets (green) and for final clusters (red). Both curves have an inflection point around cluster size 2,500 (approximately 3.4 on the *x*-axis). Note that these plots give the cumulative distribution function (cdf), while the power law exponents reported in the text are for the number of clusters of size *X* (i.e., the probability density function [pdf]). The relationship between these exponents is β_pdf_ = 1 + β_cdf_.

For the second evaluation, we selected all sequences with Pfam matches, and each sequence was assigned to the Pfam that matches it with the highest score. With this assignment, the Pfams induce a partition on the sequences. The distribution of the number of sequences in clusters induced by the Pfams was compared to those of clusters from the clustering method. Figure 12A shows comparison as a log–log plot of the number of sequences versus the number of clusters with at least that many sequences for the two cases. The plot shows that cluster size distributions are quite similar, with both the methods having an inflection point around 2,500. The difference between the two curves is that there are more big clusters (and also fewer small clusters) induced by the Pfams as compared to the clustering method. This can be explained by noting that two sequences that are in the same Pfam cluster can nevertheless be put into different clusters by the clustering method if they differ in their remaining portions.

Our clustering also shows a good correspondence with HMM profiling on the phylogenetic markers that we looked at. The clustering identifies 7,423, 12,553, and 13,657 sequences, respectively, for RecA (cluster ID 1146), Hsp70 (cluster ID 197), and RpoB (cluster ID 1187). HMM profiling identifies 5,292, 12,298, and 12,165 sequences, respectively, for these families. For each of these families, there are at least 94% of sequences (relative to the smaller set) in common between clustering and HMM profiling.

### Difference in ratio of predicted proteins to total ORFs for the PG set and the GOS set.

The ratio of clustered ORFs to total ORFs is significantly higher for the GOS ORFs (0.3471) compared to the PG ORFs (0.1888). This can be explained by the fragmentary nature of the GOS data. For the large majority of the GOS data, the average sequence length is 920 bp compared to full-length genomes for the PG data. For the PG data, clustered ORFs have a mean length of 325 aa and a median length of 280 aa. Unclustered ORFs have a mean length of 119 aa and a median length of 87 aa. Assuming that the genomic GOS data has a similar underlying ORF structure to PG data, the effect that GOS fragmentation had on ORF lengths is estimated. Each reading frame will have a mixture of clustered and unclustered ORFs, but on average there will be 2 ORFs per reading frame per 920-bp GOS fragment, and both ORFs will be truncated. Assuming the truncation point for the ORF is uniformly distributed across the ORF, the truncated ORF will drop below the 60-aa threshold to be considered as an ORF with a probability of 60/(length of the ORF). Using the median length, the percentage of clustered ORFs dropping below the threshold due to truncation is 21%; for unclustered ORFs, it is 69%. Accounting for this truncation, the expected ratio of clustered ORFs to total ORFs for the GOS ORFs based on the PG ORFs would be 0.3708, which is very close to the observed value.

### Kingdom assignment strategy and its evaluation.

We used several approaches to assign kingdoms for GOS sequences. They are all fundamentally based upon a strategy that takes into account top BLAST matches of a GOS sequence to sequences in NCBI-nr, and then voting on a majority.

We evaluated a simple strict-majority voting scheme (of the top four BLAST matches) using the NCBI-nr set. First, the redundancy in NCBI-nr was removed using a two-staged process. A nonredundant set of NCBI-nr sequences was computed involving matches with 98% similarity over 95% of the length of the shorter sequence (using the procedure discussed in Materials and Methods [Identification of nonredundant sequences]). This set was made further nonredundant by considering matches involving 90% similarity over 95% of the length of the shorter sequence. The nonredundant sequences that remained after this step constituted the evaluation dataset *S*. For each sequence in *S*, its top four BLAST matches to other sequences in *S* (ignoring self-matches) were used to assign a kingdom for it (based on a strict majority rule). This predicted kingdom assignment for the sequence was compared to its actual kingdom. A correct classification is obtained for 93% of the sequences. The correct classification rate per kingdom is given in Table 13.

**Table 13:**
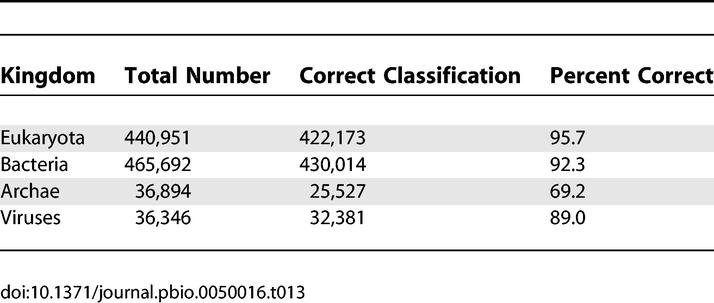
BLAST-Based Classification Rate per Kingdom

While this evaluation shows that the BLAST-based voting scheme provides a reasonable handle on the kingdom assignment problem, there are caveats associated with it. The kingdom assignment for a set of query sequences is greatly influenced by the taxonomic groups from each kingdom that are represented in the reference dataset against which these queries are being compared. If certain taxa are only sparsely represented in the reference set, then, depending on their position in the tree of life, queries from these taxa can be misclassified (using a nearest-neighbor type approach based on BLAST matches). This explains why the archaeal classification rate is quite low compared to the others. Thus, the true classification rate for the GOS dataset based on this approach will also depend on the differences in taxonomic biases in the GOS dataset (query) and the NCBI-nr set (reference).

The kingdom proportion for the GOS dataset reported in Figure 1 is based on a kingdom assignment of scaffolds. Those GOS ORFs with BLAST matches to NCBI-nr were considered, and the top-four majority rule was used to assign a kingdom to each of them. Using the ORF coordinates on the scaffold, the fraction (of bp) of a scafffold assigned to each kingdom was computed. The scaffold was labeled as belonging to a kingdom if the fraction of the scaffold assigned to that kingdom was >50%. All ORFs on this scaffold were then assigned to the same kingdom.

### Cluster size distribution, the power law, and the rate of protein family discovery.

Earlier studies of protein family sizes in single organisms [137–139] have suggested that *P*(*d*), the frequency of protein families of size *d,* satisfies a power law: that is, *P*(*d*) ≈ *d ^−^* ^β^ with exponent β reported between 2.68 and 4.02. Power laws have been used to model various biological systems, including protein–protein interaction networks and gene regulatory networks [42,43]. Figure 12B illustrates the distribution of the cluster sizes from our data on a log–log scale, a scale for which a power law distribution gives a line. In contrast to family size distributions reported in single organisms, the cluster sizes from our data are not well described by a single power law. Rather, there appear to be different power laws: one governs the size distribution of very large clusters, and another describes the rest. This behavior is observed both in the distribution of the core set sizes and also in the distribution of the final cluster sizes. We identified an inflection point for both the core set distribution and the final clusters at around size 2,500, and estimated the power law exponent β via linear regression separately in each size regime. For the core set distribution, the exponent β = 1.99 (*R* ^2^ = 0.994) for clusters of size ≤ 2,500, and β = 3.34 (*R* ^2^ = 0.996) for clusters of size > 2,500. For the final cluster sizes, the exponent β = 1.72 (*R* ^2^ = 0.995) for clusters of size ≤ 2,500, and β = 2.72 (*R* ^2^ = 0.995) for clusters of size > 2,500. The estimates for β are different for the core clusters compared to the final clusters, reflecting a larger number of medium and large clusters in the final clustering as a result of the cluster-merging and additional recruitment steps. A similar dichotomy between the size distributions of large and small protein families was observed in a study [140] of protein families contained in the ProDom, Protomap, and COG databases, where the exponent β reported was in the range of 1.83 to 1.98 for the 50 smallest clusters and 2.54 to 3.27 for the 500 largest clusters in these databases.

Our clustering method was run separately on the following seven datasets: set 1 consisted of only NCBI-nr sequences; set 2 consisted of all sequences in NCBI-nr, ENS, TGI-EST, and PG; sets 3 through 6 consisted of set 2 in combination with a random subset of 20%, 40%, 60%, and 80% of the GOS sequences, respectively; set 7 consisted of set 2 in combination with all the GOS sequences. On each of the seven datasets, the redundancy removal (using the 98% similarity filter) was run, followed by the core set detection steps. Figure 2 shows the number of core sets of varying sizes (≥3, ≥5, ≥10, and ≥20) as a function of the number of nonredundant sequences for each dataset.

The observed linear growth in number of families with increase in sample size *n* is related to the power law distribution in the following way. We model protein families as a graph where each vertex corresponds to a protein sequence and an edge between two vertices indicates sequence similarity between the corresponding proteins. Consider a clustering (partitioning) of the vertices of a graph with *n* vertices such that the cluster sizes obey a power law distribution. Let C*_d_*(*n*) [respectively, C_≥*d*_(*n*)] denote the number of clusters of size *d* (respectively, ≥*d*). Since the distribution of cluster sizes follows a power law, there exist constants α*,* β such that for all *x* ≤ *n*, *C_x_*(*n*) = α*x^−^* ^β^.

As every vertex of the graph is a member of exactly one cluster,

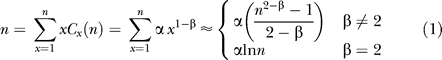
The number of clusters of size at least *d* is

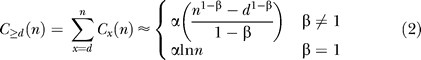
Combining the two equations, we obtain values (up to a multiplicative constant) for *C*_≥*d*_(*n*) as shown in Table 14. In all cases with β > 1, the number of clusters *C*_≥*d*_(*n*) increases as *n* increases, and as *d* decreases. Specifically, for β > 2, the growth is linear in *n* for all *d*, with slope decreasing as *d* increases. For 1 < β < 2, the growth is sublinear in *n* for all *d*.

**Table 14:**
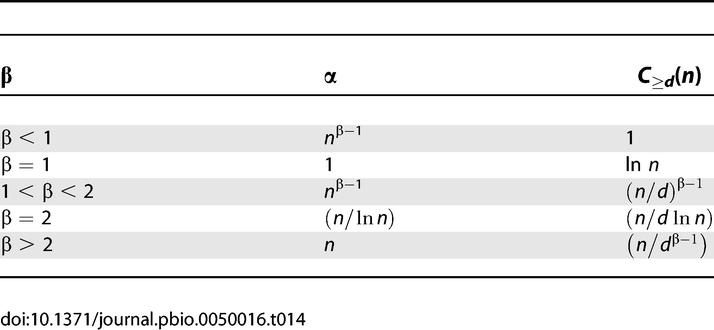
The Values for *C_≥d_*(*n*), the Number of Clusters of Size *≥d*, as a Function of the Power Law Exponent β and Constant α

Note that while the observed distribution of protein family sizes is fit by two different power laws, one for clusters of size less than 2,500 with β = 1.99 and another for clusters of size greater than 2,500 with β = 3.34 for the current number of (nonredundant) sequences, the contribution of large families to the rate of growth is negligible compared to the small families.

**Figure 13:**
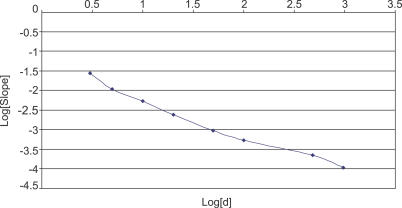
Log–Log plot of Slopes *m*(*d*) of Linear Regression Fit to the Rate of Growth in Figure 2 for Different Values of Cluster Size *d* According to the equation derived in the text, *m*(*d*) *= md^1^* ^−β^ for some constant *m*. The best linear fit to log [*m*(*d*)] gives a line with slope −0.91 (*R* ^2^ = 0.98) that is close to the predicted value 1 − β = −0.99.

The above formulas for *C*_≥*d*_(*n*) also suggest the dependence of the rate of growth of clusters on the cluster size *d*. For example, in the case when β is very close to 2,

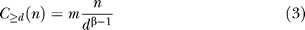
for some constant *m*. Thus, the rate of growth of cluster sizes is linear, and the slope *m*(*d*) of rate of growth is given by *m*(*d*) *= md* ^1−β^. Figure 13 shows how well the observed rates of growth match the values predicted by this equation. A fit to a sublinear function (not shown) also gives similar results as in Figure 13.

### GOS versus known prokaryotic versus known nonprokaryotic.

Examples of top five clusters in the various categories (except GOS-only) are given below. The cluster identifiers are in parentheses.

Known prokaryotic only: (Cluster ID 1319) outer surface protein in *Anaplasma ovis, Wolbachia, Ehrlichia canis;* (Cluster ID 10911) nitrite reductase in uncultured bacterium; (Cluster ID 1266) outer membrane lipoprotein in *Borrelia;* (Cluster ID 8595) methyl-coenzyme M reductase subunit A in uncultured archaeon; (Cluster ID 2959) outer membrane protein in *Helicobacter.* Known nonprokaryotic only: (Cluster ID 2226) Pol polyprotein HIV sequences; (Cluster ID 4023) maturase K; (Cluster ID 6257) NADH dehydrogenase subunit 2; (Cluster ID 8644) HIV protease; (Cluster ID 12196) MHC class I and II antigens. GOS and known prokaryotic only: (Cluster ID 3369) carbamoyl transferase; (Cluster ID 688) apolipoprotein N-acyltransferase; (Cluster ID 3726) potassium uptake proteins; (Cluster ID 300) primosomal protein N′; (Cluster ID 4605) DNA polymerase III delta subunit. GOS and known nonprokaryotic only: (Cluster ID 186) seven transmembrane helix receptors; (Cluster ID 2069) zinc finger proteins; (Cluster ID 3092) MAP kinase; (Cluster ID 1413) potential mitochondrial carrier proteins; (Cluster ID 233) pentatricopeptide (PPR) repeat-containing protein. Known prokaryotic and known nonprokaryotic only: (Cluster ID 3510) immunoglobulin (and immunoglobulin-binding) proteins; (Cluster ID 600) expansin; (Cluster ID 50) pectin methylesterase; (Cluster ID 6492) lectin; (Cluster ID 986) BURP domain-containing protein. GOS and known prokaryotic and known nonprokaryotic: (Cluster ID 2568) ABC transporters; (Cluster ID 49) short-chain dehydrogenases; (Cluster ID 4294) epimerases; (Cluster ID 1239) AMP-binding enzyme; (Cluster ID 2630) envelope glycoprotein.

### Neighbor functional linkage methods.

For the sequences in each GOS-only cluster, we determined if neighboring ORFs occurring on the same strand had a similar biological process in the GO [49]. If this shared biological process of the neighbors occurred statistically more often than expected by chance, that inferred a potential operon linkage and a biological process term for the GOS-only cluster. This approach weighted ORFs by sequence similarity to reduce the skewing effect of sequences from highly related organisms.

For definition of linked ORFs, we collected pairs of same-strand ORF protein predictions with intergenic distances less than 500 bp. Negative distances were possible if the 5′ end of the downstream ORF in the pair occurred 5′ to the 3′ end of the upstream ORF. We used a probability function to estimate the probability that two putative genes belong to the same operon given their intergenic distance [47]. Because sequences come from a variety of unknown organisms, the probability distribution was created by averaging properties of 33 randomly chosen divergent genomes. The exact choice of genomes did not greatly affect the ability of the distribution to separate experimentally determined same-operon gene pairs from adjacent, same-strand gene pairs in different known operons annotated in a version of RegulonDB downloaded on March 29, 2005 [141].

We measured the functional linkage between two protein clusters by searching for all occurrences of nearby pairs of ORFs belonging to the two clusters of interest. Sufficiently close pairs were more likely to be encoded in the same operon. We devised a scoring mechanism to reward those pairs of clusters for which many divergent examples of likely operon pairs existed in the set of ORF pairs. For each pair of clusters, a weight was applied to the contribution of each pair of ORFs, and this was proportional to how similar the pair of ORFs was to other example pairs. Thus, many near-identical pairs of ORFs, likely from the same or similar species, are not overrepresented in the final cluster pair score, while conserved examples of neighboring position from more divergent sequences contribute an increased weight. The score for each cluster pair is calculated as:

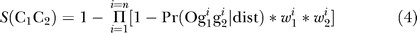
where *S*(C_1_C_2_) is the linkage score of clusters C_1_ and C_2_. The probability 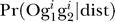 that any two genes 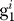 from C_1_ and 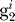 from C_2_ are in an operon is dependent on the distance between them as calculated by [47], and is weighted according to the sequence weights 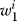 and 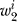 described below, for all example pairs *i*.

**Figure 14:**
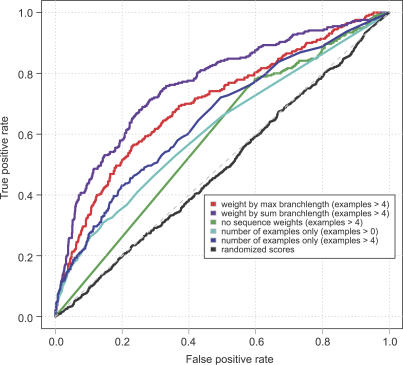
Receiver Operating Characteristic Curve Used to Evaluate Various Methods of Scoring Pairs of Clusters for Functional Similarity Pairs of clusters with ≥1 example of neighboring ORFs and assigned GO terms were divided into a set of functionally related (true positive) and functionally unrelated (true negative) cluster pairs based on the similarity of their GO terms. The scoring methods evaluated are described in the text.

We calculated sequence weights in a manner similar to that used in progressive multiple sequence alignment [142]. Briefly, neighbor-joining trees were built for all clusters using the QuickJoin [143] and QuickTree programs [144] based on a distance matrix constructed from all-against-all BLAST scores within a cluster, normalized to self-scores. For those few clusters with more than 30,000 members, trees were not built. Instead, equal sequence weights for all members were assigned because of computational limitations. The root of each tree was placed at the midpoint of the tree by using the retree package in PHYLIP [145]. The individual sequence weights were then computed by summing the distance from each leaf to the root after dividing each branch's weight by the number of nodes in the subtree below it. Weights were normalized so that the sum of weights in any given tree was equal to 1.0. This weighting scheme is superior to one in which weights are normalized to the largest weight in the tree, one that does not weight sequences according to divergence, and one that only considers the number of example pairs seen (Figure 14). To compare the different scoring methods, pairs of clusters annotated with GO terms that contained adjacent ORFs in the data were gathered. These pairs were divided into into functionally related and unrelated clusters based on a measure of GO term similarity (*p*-value ≤ 0.01) [146]. We evaluated scoring methods for the ability to recover functionally similar pairs. In all analyses, linkages between clusters were ignored if there were fewer than five examples of cluster member ORFs adjacent to each other on a scaffold.

Function for novel families was inferred as follows. (1) Assignment of GO terms to clusters. We downloaded the GO [49] database on September 21, 2005, from http://www.geneontology.org, along with the files gene_association.goa_uniprot and pfam2go.txt dated July 12, 2005. Only the biological process component of the ontology was considered. If a cluster had at least 10% of its redundant sequences annotated by the most abundant Pfam domain for that cluster, and that Pfam domain had a GO biological process term provided by the pfam2go mapping, then we assigned a cluster the GO term of its most abundant Pfam annotation. In addition, if a cluster contained at least 20% of its Uniprot GO annotations the same, it was assigned that GO term. For each cluster, redundant GO terms found on the same path to the root were removed. (2) Identification of neighbors to GOS-only clusters. Neighbors of GOS-only clusters were defined as those clusters that had a cluster linkage score above a predetermined threshold (1 × 10^−6^) and had at least five examples of cluster members adjacent to each other in the data. These neighbors were then screened for those that had been annotated with a GO term by the process described above. (3) Overrepresentation of neighbor GO terms. We attempted to define GO terms for a set of GOS-only neighbors that were statistically overrepresented. Because of the highly dependent nature of the terms in the GO, a simulation-based approach was chosen to determine which terms might be overrepresented. Annotated neighbors to a cluster of unknown function were identified as described above. For each annotated neighbor, counts for the associated GO term and all terms on the path to the root of the ontology were incremented. A total of 100,000 simulated neighbor lists of the same size as the true neighbor list were computed by selecting without replacement from those clusters with annotated GO terms, and an identical counting scheme was performed for each simulation. Overrepresentation of neighbor terms was calculated for each term on the ontology by asking how many times out of the 100,000 simulations the count for each GO term in the ontology met or exceeded the observed count for the actual neighbors. This fraction of simulations was interpreted as a *p-*value. If a term is unusually prevalent in the true observed neighbors, it should be relatively infrequent in the simulated data. For the purpose of the metric used here, “is-a” and “part-of” relationships were treated equally. In cases where a cluster had more than one GO term assigned to it, any redundant terms occurring on each other's path to the root were first removed. For any remaining clusters with nonredundant, multiple GO annotations, all possible lists of functions for each list of neighbor clusters were enumerated, and one function from each cluster was chosen. Each node in the ontology was assigned the maximum count observed from the enumerated function lists. We consistently applied this rule for the observed and simulated data.

**Figure 15:**
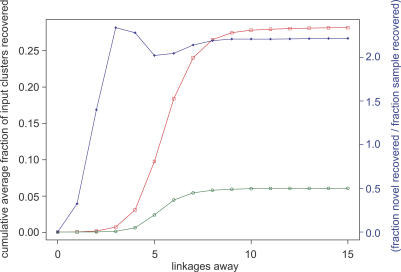
Novel GOS-Only Clusters Are More Interconnected Than a Size-Matched Sample of Clusters Red line, novel clusters; green line, size-matched sample; blue line (right axis), log_2_ ratio of fraction novel clusters recovered divided by fraction sample clusters recovered.

The following descriptive measures of the novel GOS-only cluster set were obtained. Transmembrane helix prediction was carried out with the programs TMHMM [147] and SPLIT4 [148]. GC content was calculated as (G + C)/(G + C + A + T) bases for each ORF in a cluster, and averaged for each cluster within a set. The GC content, reported as the mean and standard deviation of the cluster averages, is as follows for each cluster set: Group I, 36.7% ± 8.0%; Group II, 35.9% ± 7.9%. Group I size-matched sample, 48.8% ± 11.1%; Group II size-matched sample, 49.5% ± 11.2%; Group I viral fraction, 37.8% ± 5.1%; Group II viral fraction, 37.3% ± 4.6%. To address the interconnectivity of the novel clusters within the context of all operon linkages, we constructed a graph with clusters as nodes and inferred operon linkages (with score ≥ 1 × 10^−6^) as edges. We then asked for every node in the set of novel clusters what was the cumulative fraction of novel nodes that could be reached within a varying edge distance from the starting node. The expectation of this fraction was calculated at each distance, and the procedure was repeated for the set of size-matched clusters (Figure 15).

**Figure 16:**
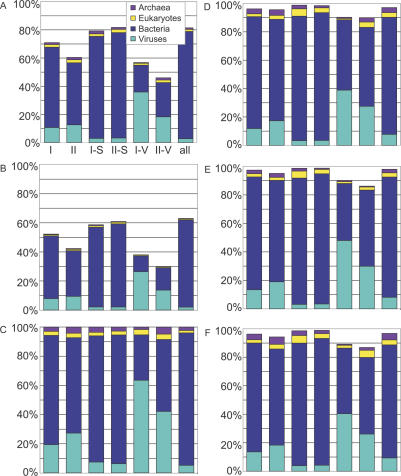
GOS-Only Clusters Are Enriched for Sequences of Viral Origin Independently of the Kingdom Assignment Method Employed For each panel, clusters are as in Figure 4. For (A–C), a kingdom is assigned to each neighboring ORF within each cluster set; the percentage of all neighboring ORFs with a given kingdom assignment is plotted. For (D–F), a kingdom is assigned to each cluster if more than 50% of all that cluster's neighbors with a kingdom assignment share the same assignment; the percentage of clusters in each set with a given assignment is plotted. In (A) and (D), a kingdom is assigned to a neighboring ORF by a majority vote of the top four BLAST matches to a protein in NCBI-nr (Materials and Methods). In (B) and (E), a kingdom is assigned if all eight highest-scoring BLAST matches agree in kingdom. In (C) and (F), all ORFs on a scaffold are assigned the same kingdom by voting among all ORFs with BLAST matches to NCBI-nr on that scaffold (Materials and Methods). In all graphs, only clusters with at least one assignable neighbor are considered. When compared to the size-matched controls, in all cases the GOS-only clusters show enrichment for viral sequences.

We tried three different BLAST-based approaches for kingdom assignment of ORFs. The first method, used in the analysis, required a majority of the four top BLAST matches to vote for the same kingdom (archaea, bacteria, eukaryota, or viruses; see Materials and Methods [Kingdom assignment strategy and its evaluation]). The second method required all eight top BLAST matches to vote for the same kingdom. The last method we used was the scaffold-based kingdom assignment described in Materials and Methods (Kingdom assignment strategy and its evaluation). Figure 16 shows the results of using these assignments to infer the kingdom of GOS-only clusters (Figure 16D–16F) and their neighboring ORFs (Figure 16A–16C). GOS-only clusters were assigned a kingdom only if >50% of their neighboring ORFs were assigned the same kingdom. The general trends observed are the same for each method, though the coverage decreases slightly for the more stringent methods.

### Characteristics and kingdom distribution of known protein domains.

For these analyses we used the predicted proteins from the public (NCBI-nr, PG, TGI-EST, and ENS) and GOS datasets. The public dataset contains multiple identical copies of some sequences due to overlaps between the source datasets. For example, many sequences in PG are also found in NCBI-nr. We filtered the public set at 100% identity to avoid overcounting these sequences. Because this filtering was necessary for the public dataset, the GOS dataset was also filtered at 100% identity. If two or more sequences were 100% identical at the residue level, but were of different lengths, only the longest sequence was kept. The resulting datasets of nonredundant proteins are referred to as public-100 and GOS-100.

We assigned each protein in public-100 to a kingdom based on the species annotations provided in the source datasets (NCBI-nr, Ensembl, TIGR, and PG). The NCBI taxonomy tree was used to determine the kingdom of each species. Of 3,167,979 protein sequences in public-100, 3,158,907 can be annotated by kingdom. The remaining 9,072 sequences are largely synthetic.

Determining the kingdom of origin of an environmental sequence can be difficult; while an unambiguous assignment can be made for some sequences, others can be assigned only tentatively or not at all. Therefore, we took a probabilistic approach (kingdom-weighting method), calculating “weights” or probabilities that each protein sequence originated from a given kingdom.

The top four BLAST matches (*E-*value < 1 × 10^−10^) of GOS ORFs to NCBI-nr were considered. The kingdom of origin for each match was determined. We pooled these “kingdom votes” for each scaffold, since (presuming accurate assembly) each scaffold must come from a single species and hence from a single kingdom. Each ORF on a scaffold contributed up to four votes. If an ORF had fewer than four BLAST matches with an *E-*value < 1 × 10^−10^, then it contributed fewer votes. ORFs with no BLAST matches contributed no votes.

In many cases, the votes were not unanimous, indicating that some uncertainty must be associated with any kingdom assignment. An additional source of uncertainty is the finite number of votes. We accounted for these statistical issues by applying the following procedure to each scaffold. First, two pseudocounts were added to the votes for the “unknown” kingdom to represent the uncertainty that remains even when votes are unanimous (especially when there are few votes). The frequency of votes for each kingdom was calculated. The vote frequency for a kingdom provides the maximum likelihood estimate of the kingdom probability (i.e., the vote frequency that would have been observed on a scaffold of similar composition but with infinitely many voting ORFs). However, that estimate may not be accurate or precise. Therefore, the multinomial standard deviation was calculated for each vote frequency *p* as SQRT [*p* × (1 − *p*)/(*n* − 1)], where *n* is the number of votes. A distance of two standard deviations from the mean corresponds roughly to a 95% confidence interval. Thus, two standard deviations were subtracted from each vote frequency, and called the result (or zero, if the result was negative) the “kingdom weight.” This “kingdom weight” is a conservative estimate. There is 95% chance that the actual kingdom probability is greater.

The kingdom weights do not sum to one because of the standard deviation penalty. The difference between the sum of the kingdom weights and unity is a measure of the total uncertainty about the kingdom assignment. This is called the “unknown weight.”

Finally, we assigned each ORF the kingdom weights calculated for the scaffold as a whole. This procedure assigned kingdom weights to many ORFs with no BLAST matches. Overall, 4,745,649 (84%) of the 5,654,638 proteins in GOS-100 receive nonzero kingdom weights.

The kingdom weights calculated in this way provide a basis for estimating the proportion of sequences originating from each kingdom, *p*_GOS_(*K*). The weights over all sequences in GOS-100 were summed for each of the known kingdoms, and divided by the sum of the weights for all kingdoms (excluding the unknown weight). This procedure suggested that 96% of the sequences are bacterial, a somewhat higher proportion than is estimated by the method described in Materials and Methods (Kingdom assignment strategy and its evaluation). Similarly, kingdom proportions, *p*_GOS–Pfam_(*K*), were calculated for the subset of GOS-100 sequences that have a significant Pfam hit, and 97% are found to be bacterial.

We used the kingdom weights directly in the analyses where possible (e.g., to calculate the expected kingdom distribution of a given set of proteins by summing the weights). However, it was necessary in some cases to use discrete assignments of a single kingdom to each ORF. A tentative assignment can be made for a given scaffold by choosing the kingdom with the highest weight. The possibility remains, in this case, that a fraction of the “unknown” weight should rightfully belong to a different kingdom. However, if a kingdom weight is greater than 0.5, then this danger is averted, and a “confident” assignment of the scaffold and its constituent ORFs to that kingdom can be made.

Given the uncertainty penalty above, achieving a kingdom weight greater than 0.5 generally requires overwhelming support for one kingdom over the others. In particular, on a given scaffold, at least eight unanimous votes for a kingdom are needed (i.e., two ORFs contributing four votes each) to make a confident assignment to that kingdom. Any disagreement between the votes increases the required number rapidly: for instance, 15 votes for a single kingdom are required to override four votes for other kingdoms.

“Confident” kingdom assignments were made for 2,626,178 (46%) of the 5,654,638 proteins in GOS-100.

In the analysis that identified new multi-kingdom Pfams, we used the subset of confidently kingdom-annotated proteins. Here, a Pfam model was designated as “kingdom-specific” in public-100 if there were only matches to proteins in one particular kingdom, and no “unknown” matches. A Pfam model that was kingdom specific in public-100 was further designated as newly “multi-kingdom” if it had matches to one or more GOS-100 proteins that were confidently labeled as belonging to a kingdom different from that found in the public-100 matches. Also, we filtered Pfam matches with an *E-*value cutoff of 1 × 10^−10^. In every case, the bit score is at least five bits greater than the trusted cutoff for the model. In addition to passing the “confident” criteria, the kingdom assignments were all confirmed by visual inspection of the BLAST kingdom vote distributions for the respective scaffolds. Because the criteria for a “confident” kingdom assignment were conservative, there were only one or a few confident assignments for each domain to a “new” kingdom. The “confident” criteria are especially difficult to meet in the case of kingdom-crossing due to the votes contributed by the crossing protein. For instance, because the IDO domain itself always contributes four votes for “Eukaryota,” at least 15 votes for “Bacteria” were required to call a scaffold “bacterial.” Thus, many scaffolds have no confident kingdom assignment.

We compared the relative diversities of protein families between GOS-100 and public-100 as represented by Pfam sequence models. In order to do this, the number of matches expected to be found for each Pfam model in the GOS-100 data was computed, assuming that the matches were distributed among the models in the same proportions that they were in the public-100 data. These “expected” match counts were compared with the observed counts to identify domains that are more diverse in GOS-100 than in public-100 and vice versa.

Because kingdoms differ in their protein usage, Pfam models match sequences from different kingdoms with different frequencies, and some models match sequences exclusively from one kingdom. Thus, to calculate the expected number of matches to a given Pfam in GOS-100 based on the number of matches observed in public-100, we corrected for the radically different kingdom composition of the two datasets.

The expected proportion of all Pfam matches in GOS-100 that are to a given model *M* was calculated as follows. First, we made a simplifying assumption that sequences from different kingdoms were equally likely to have a Pfam hit, and thus that the Pfam matches in GOS-100 would be distributed among the kingdoms according to the kingdom proportions calculated using the weighted method above (for instance, it is assumed that 97% of the matches would be to bacterial sequences). Probability that a Pfam hit in GOS-100 is from *K* ≈ *p*_GOS-Pfam_(*K*) (for sequences in GOS-100 with at least one Pfam hit) for kingdoms *K* in {Archae, Bacteria, Eukaryotes, Viruses}.

Second, we assumed that Pfam models match with the same relative rates within each kingdom in GOS-100 as they do in public-100. For instance, since twice as many SH3 domains as SH2 domains are found in public-100 eukaryotic sequences, the same ratio is expected to be found in GOS-100 eukaryotic sequences. Using the public-100 data, we calculated the frequency of matches for each Pfam model *M* within each kingdom, relative to the total number of Pfam matches to that kingdom. Pseudocounts of one were added to both the “match” and “no match” counts (i.e., using a uniform Dirichlet prior), to allow proper statistical treatment of families with few or no matches in the public databases for some kingdom. In Equation 5 below, Obs_public_(*M,K*) is the observed number of public-100 hits to *M* in *K,* and Obs_public_(*K*) is the observed number of public-100 hits to all models in *K*.

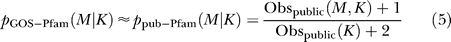



By multiplying the conditional probability of each model given a kingdom by the respective kingdom probability (*p*_GOS-Pfam_(*K*), calculated as described above in “Kingdom annotation of GOS-100 proteins: kingdom weighting method”), the proportions of Pfam matches in GOS-100 due to each combination of kingdom and Pfam model were then predicted. Finally, these predictions were summed across kingdoms to obtain the expected proportion of matches to each model.

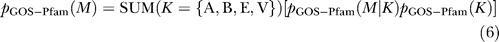



Relatively fewer GOS-100 sequences than public-100 sequences have a Pfam hit (likely because Pfam is based on sequences in the public databases). To avoid systematically overestimating the number of GOS-100 hits for each Pfam model due to this global effect, the predicted counts were based on the observed total number of Pfam matches to all models in GOS-100, and an attempt was made to predict only how these matches are distributed among models. Thus, the expected number of Pfam hits to a given model in GOS-100 is equal to the expected proportion of hits to that model, as calculated above, multiplied by the total number of Pfam hits. In the equation below, Obs_GOS_ is the total number of Pfam hits to all models in GOS-100.

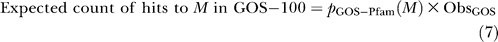



In summary, calculation of the expected number of Pfam hits to a model *M* in GOS-100 for all kingdoms can be expressed in one equation as follows:

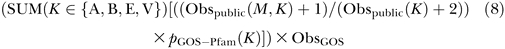
where Obs_public_(*M,K*) is the observed number of public-100 hits to model *M* in *K,* Obs_public_(*K*) is the observed number of public-100 hits to all models in *K*, *p*_GOS-Pfam_(*K*) is the proportion of GOS-100 sequences that have at least one Pfam hit in *K*, and Obs_GOS_ is the total number of Pfam hits to all models in GOS-100.

The ratio of the observed to the predicted number of hits for each Pfam model is a measure of the relative diversity of that Pfam family in GOS-100 compared to public-100, corrected for the differing kingdom proportions in the two datasets. We computed the significance of this ratio using the CHITEST function in Excel, which implements the standard Pearson's Chi-square test with one degree of freedom and expresses the result as a probability. For many protein families, the difference in diversity between the two datasets was so pronounced that Excel reports a probability of zero due to numerical underflow, indicating a *p*-value less than 1 × 10^−303^.

### IDO analysis.

The GOS-100 and public-100 sequences selected for the IDO family alignment matched the PF01231 Pfam fs model with a score above the trusted bit-score cutoff at the sequence level. In addition, the sequences were required to have the width of their matching region spanning over 50% of the Pfam IDO HMM model length. Next, all sequence matches to the Pfam IDO model from the NCBI-nr database downloaded on March 6, 2006, were added (these also satisfied the trusted score cutoff and model alignment span criteria). An additional 26 IDO sequences were found in the new sequence database relative to the GOS public sequence data freeze after filtering for identical and 1 aa different sequences and presence of first and last residues in the final trimmed alignment. Jevtrace (version 3.14) [149] was used to assess alignment quality, to remove sequences problematic for alignment, to remove sequence redundancy (at the 0-aa and 1-aa difference levels) while allowing for redundant nonoverlapping sequences, to trim the alignment to a block of aligned columns, to delete columns with more than 50% gaps, and to remove sequences with missing first or last residues. One sequence (GenBank ID 72038700) was likely a multidomain protein problematic for alignment and was removed manually. This set of procedures produced a block sequence alignment of 144 sequences and 231 characters. We aligned sequences with MUSCLE (version 3.52) [134] using default parameters. The final alignment was used to reconstruct phylogenies with a series of phylogeny reconstruction methods: PHYML [150], Tree-Puzzle [151], Weighbor [152], and the protpars program from the PHYLIP package (version 3.6a3) [145]. Bootstrapping was performed with the protpars program using 1,000 bootstrap replicates, each with 100 jumbles; the majority consensus tree was produced by the consense program in the PHYLIP package.

### Structural genomics implications.

The Pfam5000 families used in this study were chosen from among the manually curated (Pfam-A) families in from Pfam version 17. We added 2,932 families with a structurally characterized representative as of October 27, 2005, to the Pfam5000 in descending order by family size, followed by 2,068 additional families without a structurally characterized representative, in descending order by family size. Pre-GOS family size was calculated as the number of sequences in public-100 that had a match to the Pfam family. Post-GOS family size was calculated as the number of sequences in public-100 and GOS-100 that matched each family. We used the results of the HMM profiling effort (using Pfams) used for this analysis.

Coverage of GOS-100 and public-100 sequences by both versions of the Pfam5000 was measured using the subset of families in Pfam 17 that were also in Pfam 16. This was done in order to enable direct comparison of coverage results with a previous study of coverage of fully sequenced bacterial and eukaryotic genomes [73]. The versions of Pfam are similar in size (Pfam 16 contains 7,677 families, and Pfam 17 contains 7,868 families).

### Phylogeny construction for various families.

For the UVDE family, sequences were aligned using MUSCLE [134] and a tree was built using QuickTree [144].

For the PP2C family, the catalytic domain portions of the sequences were identified and aligned using the PP2C Pfam model. Sequences that contained ≥70% nongaps in this alignment were used to generate a phylogenetic tree of all the PP2C-like sequences. The phylogeny was inferred using the protdist and neighbor-joining programs in PHYLIP [145]. We used 1,941 total PP2C-like sequences for the phylogenetic analysis. The breakdown was as follows: public eukaryotic sequences, 73%; public bacterial sequences, 14%; GOS-eukaryotic sequences, 2%; GOS-bacterial sequences, 10%; and GOS-viral and GOS-unknown sequences, less than 1% combined.

For the type II GS family, sequences in GOS and NCBI-nr were searched with a type II GS HMM constructed from 17 previously known bacterial and eukaryotic type II GS sequences. Matching sequences from NCBI-nr and GOS were filtered separately for redundancy at 98% identity; the combined set of sequences was aligned and a neighbor-joining tree was constructed.

For the RuBisCO family, matching RuBisCO sequences from GOS and NCBI-nr were filtered separately for redundancy at 90% identity, resulting in 724 sequences in total. The 724 RuBisCO sequences were then aligned and a neighbor-joining tree was constructed.

### Identification of proteases.

**Figure 17:**
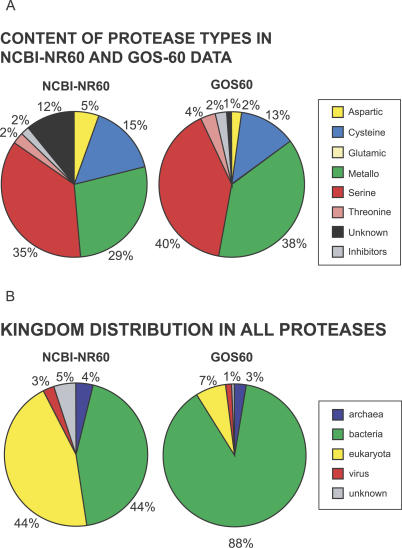
Content of Protease Types in NCBI-nr and GOS, and Kingdom Distribution of All Proteases Due to the highly redundant nature of some NCBI-nr protease groups, nonredundant sets for both NCBI-nr and GOS are computed; these nonredundant sets are referred to as NCBI-nr60 and GOS60.

**Figure 18:**
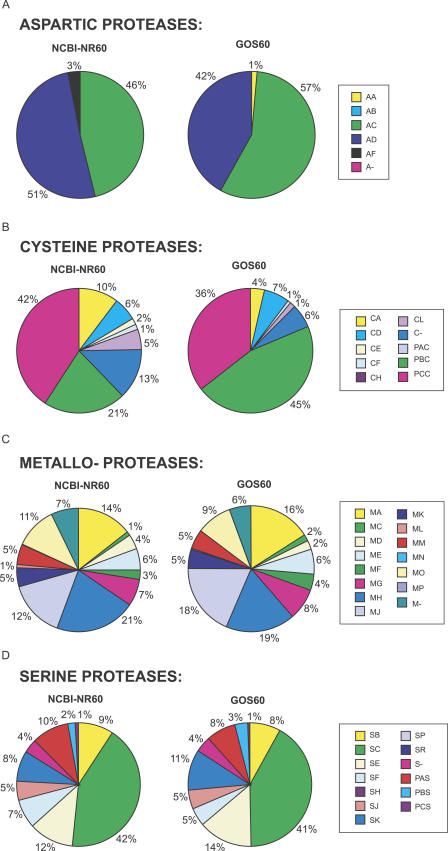
Content of Bacterial Protease Clans

We clustered sequences in the MEROPS Peptidase Database [100] using CD-HIT [116,117] at 40% similarity level. This resulted in 7,081 sequences, which were then divided into groups based on catalytic type and Clan identifier. These sequences were used as queries to search against a clustered version of NCBI-nr (clustered at 60% similarity threshold) using BLASTP (*E-*value ≤ 1 × 10^−10^). A similar search was carried out against GOS (clustered at 60% similarity threshold). Figure 17 shows the content of protease types in NCBI-nr and GOS together with the kingdom distributions. Figure 18 shows the content of bacterial protease clans.

### Metabolic enzymes in GOS.

Hmmsearch from the HMMER package [105] was used to search the GOS sequences for different GS types. The GlnA TIGRFAM model was used for finding GSI sequences. The HMMs built from known examples of 17 GSII and 18 GSIII sequences from NCBI-nr were used to search the GOS sequences.

### Identification of ORFans in NCBI-nr.

ORFans are proteins that do not have any recognizable homologs in known protein databases. A straightforward way to identify ORFans is through all-against-all sequence comparison using relaxed match parameters. However, this is not computationally practical. An effective approach is to first remove the non-ORFans that can be easily found, and then to identify ORFans from the remaining sequences.

We identified non-ORFans by clustering the NCBI-nr with CD-HIT [116,117], an ultrafast sequence clustering program. A multistep iterated clustering was performed with a series of decreasing similarity thresholds. NCBI-nr was first clustered to NCBI-nr90, where sequences with >90% similarities were grouped. NCBI-nr90 was then clustered to NCBI-nr80/70/60/50 and finally NCBI-nr30. After each clustering stage, the total number of clusters of NCBI-nr was decreased and non-ORFans were identified. A one-step clustering from NCBI-nr directly to NCBI-nr30 can be performed. However, the multistep clustering is computationally more efficient.

At the 30% similarity level, all the NCBI-nr proteins were grouped into 391,833 clusters, including 259,571 singleton clusters. The proteins in nonsingleton clusters are by definition non-ORFans. However, proteins that remain as singletons are not necessarily ORFans, because their similarity to other proteins may not be reported for two reasons: (1) significant sequence similarity can be <30%; and (2) in order to prevent a cluster from being too diverse, CD-HIT, like all other clustering algorithms, may not add a sequence to that cluster even if the similarity between this sequence and a sequence in that cluster meet the similarity threshold.

The 259,571 singletons were compared to NCBI-nr with BLASTP [38] to identify real ORFans from them. The default low-complexity filter was enabled in the BLAST comparisons, and similarity threshold in the form of an *E-*value was set to 1 × 10^−6^. In the end, 84,911 proteins with at least 100 aa are identified as ORFans. About 100,000 short ORFans less than 100 aa were removed from this study, because they may not be real proteins.

### Genome sequencing projects and rate of discovery.

We used Ensembl sequences for *Homo sapiens, Mus musculus, Rattus norvegicus, Canis familiaris,* and *Pan troglodytes.* Their clustering information is shown in Table 15. When we considered the datasets in the order HS, HS + MM, HS + MM + RN, HS + MM + RN + CF, and HS + MM + RN + CF + PT, the numbers of distinct clusters were 10,536, 12,731, 13,605, 14,606, and 14,993, respectively. These numbers were compared against a random subset of NCBI-nr bacterial sequences (of a similar size) and also against a random subset of GOS sequences. We also randomized the order of the mammalian sequences to produce a dataset that was independent of the genome order being considered.

**Table 15:**
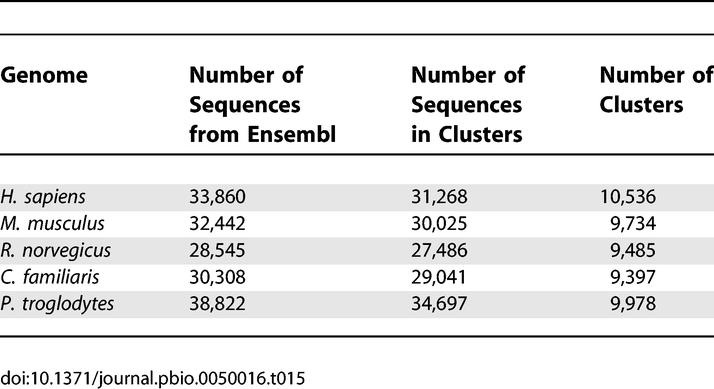
Clustering Information for Ensembl Sequences for *H. sapiens, M. musculus, R. norvegicus, C. familiaris*, and P. troglodytes

## Supporting Information

Protocol S1 Supplementary Information (25 KB DOC)

### Accession Numbers

All NCBI-nr sequences from February 10, 2005 were used in our analysis. Protocol S1 lists the GenBank (http://www.ncbi.nlm.nih.gov/Genbank) accession numbers of (1) the genomic sequences used in the PG set, (2) the sequences used in building GS profiles, and (3) the NCBI-nr sequences used in building the IDO phylogeny. The other GenBank sequences discussed in this paper are Bacillus sp. NRRL B-14911 (89089741), Janibacter sp. HTCC2649 (84385106), Erythrobacter litoralis (84785911), and Nitrosococcus oceani (76881875). The Pfam (http://pfam.cgb.ki.se) structures discussed in this paper are envelope glycoprotein GP120 (PF00516), reverse transcriptase (PF00078), retroviral aspartyl protease (PF00077), bacteriophage T4-like capsid assembly protein (Gp20) (PF07230), major capsid protein Gp23 (PF07068), phage tail sheath protein (PF04984), IDO (PF01231), poxvirus A22 protein family (PF04848), and PP2C (PF00481). The glutamine synthetase TIGRFAM (http://www.tigr.org/TIGRFAMs) used in the paper is GlnA: glutamine synthetase, type I (TIGR00653). The PDB (http://www.rcsb.org/pdb) identifiers and the names of the eight PDB ORFans with GOS matches are: restriction endonuclease MunI (1D02), restriction endonuclease BglI (1DMU), restriction endonuclease BstYI (1SDO), restriction endonuclease HincII (1TX3); alpha-glucosyltransferase (1Y8Z), hypothetical protein PA1492 (1T1J), putative protein (1T6T), and hypothetical protein AF1548 (1Y88).

## Abbreviations

aa: amino acid
ENS: Ensembl
EST: expressed sequence tag
GO: Gene Ontology
GOS: Global Ocean Sampling
GS: glutamine synthetase
HMM: hidden Markov model
IDO: indoleamine 2,3-dioxygenase
NCBI: National Center for Biotechnology Information
ORF: open reading frame
PDB: Protein Data Bank
PG: prokaryotic genomes
PP2C: protein phosphatase 2C
PSI: Protein Structure Initiative
RLP: RuBisCO-like protein
TGI: TIGR gene indices
TC: trusted cutoff
UVDE: UV dimer endonuclease
