# Supplementary material for: The Sorcerer II Global Ocean Sampling Expedition: Expanding the Universe of Protein Families
Source: PLoS Biol. 2007 Mar 13;5(3):e16. doi: 10.1371/journal.pbio.0050016 (PMC1821046; doi:10.1371/journal.pbio.0050016)
Supplement: Protocol S1 — (25 KB DOC) [file pbio.0050016.sd001.doc]

**SUPPLEMENTARY INFORMATION**

**PG ORF data set**: ORFs were predicted on sequences from 222 prokaryotic genomes. Sequences with the following GenBank accession identifiers (gi’s) were used: 49529273, 47118298, 17922003, 16445345, 56387602, 6626248, 6626247, 56311475, 50082967, 20520073, 30260185, 49176966, 42740913, 29899096, 51973633, 47118318, 56160984, 52346357, 56908016, 38680335, 49328240, 52214156, 29342101, 49237636, 49239191, 42494925, 58012118, 33591071, 33591070, 33591069, 2687879, 51572834, 47118316, 17986242, 54112365, 47118299, 27904416, 21672292, 52421840, 52208053, 57165696, 30407139, 33518905, 19172958, 29251571, 12057206, 29835126, 12057210, 6626250, 47118320, 33236960, 21672293, 34105712, 25168256, 47118322, 28204652, 38231477, 47118314, 42602314, 29542685, 57223978, 11612676, 50874889, 46451220, 58416339, 57160810, 58417290, 29350190, 49609491, 26111730, 48994873, 47118301, 56384585, 56603679, 20095250, 56378377, 39985517, 37508091, 58000905, 33149228, 6626252, 55229667, 12057215, 32263428, 6626253, 12057207, 56178122, 58253671, 41584196, 30407129, 13400022, 53752796, 53749768, 52627367, 50950407, 45602555, 24204616, 30407126, 30407125, 46882319, 52306107, 50363371, 47118328, 6626255, 45050763, 19913449, 19918815, 20986624, 6626257, 53756053, 41400296, 31742509, 30407142, 50952454, 41353971, 31541778, 6626254, 53987142, 47458799, 42494967, 47118307, 26117688, 30407144, 40068520, 12057208, 30407145, 30407130, 54013472, 47118302, 42632302, 39721595, 46399275, 13400023, 47419843, 37510968, 48429720, 34398108, 33772318, 33238865, 33772317, 50839098, 12057214, 24987239, 28856110, 18308975, 30407140, 18980902, 47118297, 30407127, 32456059, 39748133, 25307999, 30407152, 51459527, 56126533, 29140506, 30407157, 16445344, 24371479, 30043918, 24080789, 56676665, 30407155, 57284222, 49240382, 49243355, 47118312, 47208328, 47118324, 27316888, 57636010, 22535226, 30407156, 24378526, 25307955, 25168257, 14286347, 50902420, 21905618, 19913450, 47118313, 55737978, 55736088, 57546753, 30407153, 25307963, 47118305, 51854827, 56684969, 33772316, 47118304, 20671658, 57158259, 30407158, 47118327, 47118315, 12057205, 46197919, 55771382, 41821838, 6626258, 30407160, 28476818, 32949295, 12057212, 47118310, 27362705, 37509034, 47118325, 42410857, 58418577, 34495246, 21240769, 21166373, 58424217, 12057211, 28058986, 30407161, 22002119, 45438631, 51587641, 56542470.

**TGI-EST data set**: The EST assemblies available from TIGR Gene Index for the following 72 organisms were downloaded - Aedes aegypti, Allium cepa, Amblyomma variegatum, Arabidopsis thaliana, Aspergillus flavus, Aspergillus nidulans, Astatotilapia burtoni, Bos taurus, Brassica napus, Brugia malayi, Canis familiaris, Capsicum annuum, Chlamydomonas reinhardtii, Ciona intestinalis, Coccidioides posadasii, Cryptosporidium parvum, Dictyostelium discoideum, Eimeria tenella, Filobasidiella neoformans, Fundulus heteroclitus, Glycine max, Gossypium, Haplochromis chilotes, Haplochromis sp red tail sheller, Helianthus annuus, Hordeum vulgare, Ictalurus punctatus, Lactuca sativa, Leishmania, Lotus japonicus, Lycopersicon esculentum, Magnaporthe grisea, Medicago truncatula, Mesembryanthemum crystallinum, Neospora caninum, Neurospora crassa, Nicotiana benthamiana, Nicotiana tabacum, Onchocerca volvulus, Oncorhynchus mykiss, Oryza sativa, Oryzias latipes, Petunia hybrida, Picea, Pinus, Plasmodium berghei, Plasmodium falciparum, Plasmodium vivax, Plasmodium yoelii, Poplar, Rhipicephalus appendiculatus, Saccharomyes cerevisiae, Saccharum officinarum, Salmo salar, Sarcocystis neurona, Schistosoma mansoni, Schizosaccharomyces pombe, Secale cereale, Solanum tuberosum, Sorghum bicolor, Sus scrofa, Tetrahymena thermophila, Theobroma cacao, Toxoplasma gondii, Trichomonas vaginalis, Triticum aestivum, Trypanosoma brucei, Trypanosoma cruzi, Vitis vinifera, Xenopus laevis, Xenopus tropicalis, and Zea mays.

The gi numbers for the NCBI-nr sequences used in constructing the IDO phylogeny:

88176990, 85374035, 85093615, 85091830, 83773646, 83768179, 77165186, 76679369, 76655955, 73979259, 72005928, 71019497, 70998959, 70993992, 688388, 68483788, 67524103, 67522803, 6322538, 62662914, 57097759, 55630586, 50806262, 50557000, 50426921, 50259424, 50199502, 50199500, 49903322, 49657731, 47215308, 46120362, 46111551, 4504577, 44981664, 3582432, 3582430, 29612616, 22122379, 13027384.

The gi numbers for sequences used for building type II GS HMMs:

33504515, 22136142, 19605, 699623, 22749655, 758150, 2388925, 45825350, 45825360, 12597977, 6636073, 32474726, 17223660, 37496559, 17936298, 121346, 121339.

The gi numbers for sequences used for building type III GS HMMs:

46579669, 56751347, 9651982, 63028437, 2895904, 15025687, 46400515, 19387550, 15073018, 17937919, 56696457, 37522068, 71084004, 13475188, 50085607, 71556049, 39934475, 399549.
